# Herbal or conventional medicine? A cross-sectional study of Jordanian university students’ preferences for cold and flu treatment

**DOI:** 10.1371/journal.pone.0358179

**Published:** 2026-09-15

**Authors:** Mohammad Al-magableh, Abdulrhman H.M. Muhaisen, Rama M. Ateir, Shatha A. Abu_Eraq, Hanan O. Hamed, Sama M. Al Atoum, Reem Bani Amer, Soha S. AlShirah

**Affiliations:** 1 Department of Pediatrics, Family Medicine, and Gynecology, Faculty of Medicine, Yarmouk University, Irbid, Jordan; 2 Department of Basic Medical Sciences, Faculty of Medicine, Yarmouk University, Irbid, Jordan; Endeavour College of Natural Health, AUSTRALIA

## Abstract

**Background:**

University students frequently self-manage common cold and influenza symptoms using herbal remedies, pharmaceutical treatments, or both. However, evidence regarding treatment preferences among Jordanian university students remains limited.

**Objective:**

To assess treatment preferences and factors associated with four outcomes: first-line treatment choice, timing of treatment initiation, response to perceived herbal treatment failure, and medication-obtaining behavior.

**Methods:**

An observational cross-sectional study was conducted in Jordan among 1,797 undergraduate students from 16 April to 16 September 2025 using a confidential, de-identified Arabic online questionnaire. Descriptive statistics and multinomial logistic regression were used to evaluate the four outcomes. Adjusted odds ratios (aORs) with 95% confidence intervals (CIs) were reported.

**Results:**

Combined therapy was the most common first-line choice (44.5%), and 42.5% initiated treatment on the same day. Female participants had higher odds of preferring combined therapy than males (aOR = 2.55, 95% CI: 1.64–3.96; p < 0.001), whereas medical-field students had lower odds of same-day treatment initiation (aOR = 0.55, 95% CI: 0.42–0.72; p < 0.001). After perceived herbal treatment failure, 50.7% switched to pharmaceutical treatments; reliance on doctor-prescribed treatments was associated with lower odds of continuing herbal remedies (aOR = 0.38, 95% CI: 0.27–0.51; p < 0.001) or combining herbal and pharmaceutical treatments (aOR = 0.56, 95% CI: 0.45–0.70; p < 0.001). Regarding medication-obtaining behavior, 28.2% purchased pharmaceutical treatments without a prescription based on personal experience, and medical-field students had higher odds of this behavior (aOR = 1.76, 95% CI: 1.39–2.23; p < 0.001).

**Conclusions:**

Treatment decisions reflected frequent combined self-care and were associated with sex, field of study, symptoms, prescription-related behavior, and treatment beliefs. Targeted health education may support more informed and rational self-care among university students.

## Introduction

Common cold and influenza are among the most common acute viral respiratory illnesses worldwide and continue to impose a substantial burden on individuals and health systems because of their high frequency, ease of transmission, and impact on daily functioning [1–3]. In addition to causing discomfort and temporary functional impairment, these conditions are associated with healthcare visits, missed study or work days, and widespread reliance on self-care strategies and over-the-counter symptom-relief products [4–7]. Because these illnesses are usually self-limiting in otherwise healthy individuals, many people make treatment decisions independently, often without formal medical consultation.

In this context, self-management of common cold and influenza symptoms is shaped not only by symptom severity but also by personal beliefs, cultural norms, prior experiences, treatment accessibility, expected speed of relief, and concerns about adverse effects [8]. Pharmaceutical treatments and over-the-counter products are commonly used for symptom relief because they are accessible and are perceived to provide rapid relief; however, inappropriate medication use, particularly antibiotic use for viral illnesses, remains a public health concern. Commonly used pharmaceutical treatments for cold and influenza symptoms include analgesics and antipyretics such as paracetamol and ibuprofen, antihistamines, decongestants, cough suppressants, and throat lozenges. In contrast, herbal remedies remain widely used because they are familiar, accessible, culturally accepted, and often perceived as safer or more “natural” options [9,10]. Examples of commonly used herbal remedies in the regional context include sage, chamomile, anise, ginger, mint, thyme, lemon, and honey-based preparations. Beyond individual preference, broader evidence also suggests that medicine choice is influenced by social and cultural factors, while contemporary studies continue to document substantial use of over-the-counter products for self-medication in cold- and cough-related symptoms [11,12].

The issue may be particularly relevant among university students, a group in which self-medication is highly prevalent [13]. Students commonly manage minor illnesses independently, and their decisions may be influenced by peer advice, social media, prior experience, academic background, and ease of access to pharmacies or home remedies. Prior studies among university populations have shown that mild symptom perception and previous personal experience are major drivers of self-medication behaviors [14–16]. In Jordan, available evidence also suggests that students frequently self-medicate when experiencing common cold and influenza symptoms rather than relying solely on physician-directed care [13,16]. Together, these findings indicate that treatment choices in this population are likely shaped by a combination of convenience, beliefs, and informal health practices.

The regional context is also important. In Jordan and the wider Middle Eastern context, herbal remedies have deep cultural roots and remain integrated into everyday health practices. The region contains rich medicinal plant diversity, and a substantial number of plant species continue to be used in Arab traditional medicine [17]. This cultural familiarity may contribute to continued reliance on herbal remedies, either alone or alongside pharmaceutical treatments, especially for common, self-limiting illnesses such as cold and influenza. At the same time, strong reliance on self-directed care raises concerns about inappropriate medication practices, including the misuse of antibiotics for viral illnesses, which represents an important public health issue in Jordan and contributes to broader antimicrobial-resistance concerns [18,19].

Despite the clinical and public health relevance of these behaviors, evidence from Jordan remains limited regarding how university students choose between herbal remedies, pharmaceutical treatments, and combined therapy when managing common cold and influenza symptoms. Previous studies among university students have examined the prevalence, patterns, attitudes, symptoms, reasons, and sources of advice related to self-medication [13,14,16]. The present study extends this literature by evaluating four specific treatment-decision domains: first-line treatment choice, timing of treatment initiation, responses to perceived herbal treatment failure, and consultation patterns before pharmaceutical treatment use. Understanding these patterns is important because they may reflect not only individual treatment beliefs but also broader issues related to health literacy, rational medicine use, and appropriate help-seeking behavior.

Therefore, this study aimed to examine treatment preferences among Jordanian university students for managing common cold and influenza symptoms, including preferences for herbal remedies, pharmaceutical treatments, and combined therapy. The study also assessed demographic, clinical, and belief-related factors associated with first-line treatment choice, timing of treatment initiation, responses to perceived herbal treatment failure, and medication-obtaining behavior. This study focused on treatment preferences, beliefs, and self-reported behaviors; it did not aim to objectively assess students’ knowledge of common cold, influenza, herbal remedies, pharmaceutical treatments, or antibiotic use.

## Methods

***Study design and participants*** – This study employed an observational cross-sectional design to assess treatment preferences among university students in Jordan for common cold and influenza symptoms. The study was designed and reported following the STROBE (Strengthening the Reporting of Observational Studies in Epidemiology) guidelines [20] for cross-sectional studies to ensure methodological rigor, transparency, and completeness in reporting.

***Eligibility criteria*** – Eligible participants were Jordanian university students aged ≥18 years who were formally enrolled in an undergraduate program at the time of participation. Students expected to graduate, defined in the recruitment materials as students who remained formally enrolled and had six or fewer credit hours remaining, were considered eligible. Responses were excluded if the participant was < 18 years, had already graduated and was no longer enrolled, or was otherwise not currently enrolled as a university student.

***Sample size calculation –*** An a priori power analysis was conducted before data collection using G*Power 3.1.9.7 (Heinrich Heine University, Düsseldorf, Germany). The calculation was based on a linear multiple regression model (fixed model, R² deviation from zero). Assuming an alpha level of 0.05, a statistical power of 0.99, a small effect size (f² = 0.02), and 10 predictors, the minimum required sample size was 1,628 participants. The analysis yielded an actual power of 0.99. The final analytical sample included 1,797 participants, exceeding the minimum required sample size.

***The Questionnaire*** – The research team developed a closed-ended questionnaire in Arabic. It was designed to take approximately five minutes to be completed by participants. The questionnaire underwent expert review by a family medicine specialist to assess face and content validity. The questionnaire was pilot-tested on 10 eligible students to assess clarity, comprehensibility, and completion time. Minor wording refinements were made before full-scale distribution. Data obtained during the pilot phase were not included in the final analysis. The questionnaire consisted of five main sections; each section required completion before participants could proceed to the next section.

1. Demographic and personal information: this section collected data on sex, age, place of residence, marital status, university, field of study (medical or non-medical), academic year, academic performance, chronic disease status, and income. The academic-year item included an “expected to graduate” category, defined in the recruitment materials as students who remained formally enrolled and had six or fewer credit hours remaining. In the English-translated questionnaire, this category was imprecisely labeled as “Graduated”; however, it did not refer to individuals who had already completed their undergraduate degree. The income item was presented using categories in Jordanian dinars but did not specify whether it referred to personal income, family financial support, income from part-time employment, or household income. Because this item could not be interpreted reliably, it was excluded from all statistical analyses.
2. Individuals’ attitudes and choices in managing common cold and influenza symptoms: this section included four questions assessing the symptoms that usually prompt participants to seek treatment, their usual timing of treatment initiation after symptom onset, their usual first-line treatment choice, and the reasons underlying that choice. These questions were intended to assess participants’ general self-reported practices when experiencing common cold or influenza symptoms, rather than behaviors related to a specific recent or medically confirmed episode.
3. The use of herbal remedies: this section included five questions about students’ preferences for using herbal remedies in case of illness, what type of herbal remedies the students prefer to use in case of common cold and influenza, and why they use them. Participants who did not prefer herbal remedies selected the option “I do not prefer using herbal remedies.”
4. The use of pharmaceutical treatments: This section included five questions for participants who preferred pharmaceutical treatments, addressing consultation behavior, commonly used treatment types, reasons for use, and concerns about pharmaceutical treatment use. Medication-obtaining behavior was assessed using predefined questionnaire response options that captured the main patterns relevant to pharmaceutical treatment use in this study, including doctor consultation and prescription-based use, purchasing pharmaceutical treatments without a prescription based on personal experience, consulting only a pharmacist, and not preferring pharmaceutical treatments. These categories were used to describe selected medication-obtaining patterns and were not intended to capture all possible sources of treatment advice, such as family members, friends, or online sources.
5. Beliefs and attitudes toward pharmaceutical treatments and herbal remedies: This section used a five-point Likert scale (strongly agree, agree, neutral, disagree, and strongly disagree). It included ten statements: the first four assessed prior experiences and social influences related to treatment choice, the next three assessed beliefs about the effectiveness of pharmaceutical treatments and herbal remedies, and the final three assessed beliefs regarding treatment safety. In addition, this section included one question about the sources from which students obtained information on the effectiveness of herbal remedies and pharmaceutical treatments for managing the common cold and influenza. The questionnaire used predefined closed-ended response options. A free-text “Other (please specify)” option was not provided for some multiple-response items, including the types of herbal remedies and pharmaceutical treatments used, motivations for herbal remedy use, factors influencing pharmaceutical treatment choice, concerns about pharmaceutical treatments, and sources of treatment information.

For the purpose of this study, perceived herbal treatment failure was operationally defined as the participant’s self-reported perception that herbal remedies did not achieve the desired symptom relief for common cold or influenza symptoms. This was assessed through the questionnaire item asking participants what they would do if natural herbal remedies did not achieve the desired results. Therefore, treatment failure in this study reflects perceived lack of effectiveness rather than a clinically confirmed treatment failure.

A preliminary exploratory factor analysis (EFA) was conducted for the initial 10 Likert-scale belief items using principal axis factoring with direct oblimin rotation. Two items were excluded based on psychometric performance, resulting in a refined 8-item, two-factor solution that explained 59.9% of the total variance. Sampling adequacy was good (KMO = 0.87), and internal consistency was acceptable to good for the two retained subscales (Cronbach’s alpha = 0.83 and 0.66). Detailed EFA and internal consistency results are provided in Supplementary Tables S2 and S5 in S6 File.

***Data collection procedure –*** Data were collected using a closed-ended Arabic questionnaire developed in Google Forms. The questionnaire was distributed via social media platforms. A QR code linked to the questionnaire was also distributed directly to eligible Jordanian university students by the research team. Participants were recruited using a convenience sampling approach from multiple Jordanian universities; therefore, the sample was not designed to be proportionally representative of all universities in Jordan. Participation was voluntary. Informed consent was obtained electronically from all participants before survey initiation. In the first section of the Google Forms questionnaire, participants were presented with an information and consent statement explaining the study purpose, voluntary nature of participation, confidentiality of responses, and de-identified data collection. Only participants who selected “I agree” were able to proceed to the questionnaire and submit responses. No written signed consent was collected because the survey was conducted online. Responses were collected in a confidential, de-identified format. To prevent duplicate submissions, the Google Forms setting “Limit to 1 response” was enabled, which required sign-in with a Google account; no personally identifying information, including email addresses, was collected or stored by the research team. Data collection took place in Jordan from 16 April 2025 to 16 September 2025.

***Analysis*** – Data were exported from Google Forms into Microsoft Excel and subsequently analyzed using IBM SPSS Statistics (version 30). Descriptive statistics were generated for participant characteristics, treatment-related outcomes, and all independent variables included in the regression models. Categorical variables, including symptom categories, medication-related concerns, consultation behaviors, and individual belief items, were summarized using frequencies and percentages. Age was summarized using the mean and standard deviation. Multinomial logistic regression models were used to identify factors associated with treatment-related choices for common cold and influenza. Four main outcomes were examined:

1. First-line treatment choice for common cold and influenza (herbal remedies, pharmaceutical treatments, or combined therapy).
2. Timing of treatment initiation (same day, after 1–2 days, or after ≥3 days).
3. Response after perceived herbal treatment failure, defined as the participant’s perception that herbal remedies did not achieve the desired symptom relief.
4. Medication-obtaining behavior (self-experience, pharmacist consultation, or not preferring pharmaceutical treatments).

For the multinomial logistic regression models, the reference outcome categories were defined a priori as follows: “Do not use any treatment” for first-line treatment choice, “Do not use any treatment” for timing of treatment initiation, “switching to pharmaceutical treatments” for response after perceived herbal treatment failure, and “doctor consultation and prescription-based use” for medication-obtaining behavior. For categorical predictors, the reference categories were specified in the corresponding regression tables, including male sex, urban residence, non-medical field of study, absence of chronic disease, absence of the reported symptom, strongly agree for Likert-scale belief items, and Yarmouk University for the institutional grouping variable.

Candidate predictors for each multinomial logistic regression model were selected based on their conceptual relevance to the corresponding outcome, previous literature, and the structure of the questionnaire. Core sociodemographic variables, including age, sex, residence, field of study, chronic disease status, and university group, were included when applicable. Outcome-specific predictors, such as symptom type, concerns about pharmaceutical treatments, consultation behavior, and belief-related items, were included only when directly relevant to the outcome being examined. For the treatment-initiation model, the item “I believe that herbal remedies can be a complete alternative to pharmaceutical treatments” was included because it most directly represented a general treatment belief that could influence whether participants initiated treatment promptly or delayed treatment. The remaining belief items addressed distinct constructs, including social influence, physician recommendation, comparative effectiveness and safety, medication avoidance, and potential adverse reactions, and were therefore not included in the treatment-initiation model. No automated stepwise variable-selection procedure was used.

Adjusted odds ratios (aORs), 95% confidence intervals (CIs), and p-values were reported. A p-value < 0.05 was considered statistically significant. Cross-tabulation and χ² tests assessed associations between sex and treatment-related behaviors.

A sensitivity analysis was performed by repeating the first-line treatment-choice multinomial logistic regression model after excluding participants from Yarmouk University, given its overrepresentation in the original sample. Additionally, an institutional grouping variable (other universities vs Yarmouk University) was included in the multinomial logistic regression models to assess whether the observed associations were influenced by the overrepresentation of a single institution.

***Ethics*** – Ethical approval was obtained on 15/04/2025 from the Institutional Review Board of Yarmouk University (IRB/2025/164). Participation was voluntary. Informed electronic consent was obtained from all participants before participation through the first section of the online questionnaire. Responses were collected confidentially in a de-identified format and were used solely for research purposes.

## Results

***Participants’ Characteristics*** – A total of 1,844 submissions were received. Forty-seven responses were excluded because the respondents were younger than 18 years, had already completed their undergraduate degree and were no longer enrolled, or were otherwise not currently enrolled university students, leaving 1,797 responses for analysis. The mean age of participants was 20.87 ± 3.02 years. Most participants were female (69.9%), lived in urban areas (62.7%), and did not report chronic illness (74.6%). Participants were almost equally distributed between medical (49.4%) and non-medical (50.6%) fields. Participant characteristics and descriptive summaries of treatment-related outcomes are presented in Table 1. Descriptive statistics for all independent variables included in the multinomial logistic regression models are provided in Supplementary S3 Table in S6 File.

**Table 1:** Participant characteristics and descriptive summary of common cold and influenza treatment-related outcomes.

No.	Variable	Category	n	%
**1**	**Total participants**	Overall sample	1,797	100.0
**2**	**Sex**	Female	1,257	69.9
		Male	540	30.1
**3**	**Residence**	Urban	1,127	62.7
		Rural	670	37.3
**4**	**Chronic illness**	Suffering	456	25.4
		Non-suffering	1,341	74.6
**5**	**Field of study**	Medical	887	49.4
		Non-medical	910	50.6
**6**	**University name**	Yarmouk University	1,138	63.3
		Jordan University of Science and Technology	181	10.1
		Al-Albayt University	156	8.7
		Al-Balqa Applied University	89	5.0
		University of Jordan	69	3.8
		Hashemite University	69	3.8
		Mu’tah University	33	1.8
		Private universities	62	3.5
**7**	**Timing of treatment initiation for common cold or influenza symptoms**	On the same day	764	42.5
		After 1–2 days	533	29.7
		After 3 days or more	136	7.6
		Do not use any treatment	364	20.3
**8**	**First-line treatment choice for common cold or influenza symptoms**	Herbal remedies	476	26.5
		Pharmaceutical treatments	375	20.9
		Combined therapy	800	44.5
		Do not use any treatment	146	8.1
**9**	**Self-reported antibiotic use for common cold or influenza symptoms**	Yes	867	48.2
		No	930	51.8

***Note.*** *Percentages are based on N = 1,797 and are presented to one decimal place. Minor discrepancies in summed percentages may occur because of rounding.*

***First-line treatment choice for Common Cold and Influenza*** – In multinomial logistic regression (Table 2), sex was independently associated with first-line treatment choice preference for common cold and influenza. Females exhibited higher odds of preferring herbal remedies (aOR = 1.87, 95% CI: 1.23–2.86; p = 0.004), pharmaceutical treatments (aOR = 1.61, 95% CI: 1.01–2.55; p = 0.044), and combined therapy (aOR = 2.55, 95% CI: 1.64–3.96; p < 0.001) relative to males. Rural residence was associated with lower odds of preferring pharmaceutical treatments (aOR = 0.57, 95% CI: 0.36–0.89; p = 0.013) and combined therapy (aOR = 0.61, 95% CI: 0.40–0.93; p = 0.023), with no significant association for herbal preference (aOR = 0.78, 95% CI: 0.52–1.17; p = 0.231). Symptom type showed limited effects; sore throat was associated with higher odds of combined therapy (aOR = 1.83, 95% CI: 1.16–2.87; p = 0.009), while fever demonstrated a borderline inverse association with herbal preference (aOR = 0.69, 95% CI: 0.45–1.04; p = 0.076). Regarding medication-related concerns, reporting “possible side effects” was associated with increased odds of preferring herbal remedies (aOR = 1.60, 95% CI: 1.03–2.49; p = 0.038), whereas reporting “no concerns” was associated with reduced odds (aOR = 0.43, 95% CI: 0.24–0.77; p = 0.005). Age, field of study, and chronic disease status were not significantly associated with treatment preference (all p > 0.05).

**Table 2:** Multinomial logistic regression for first-line treatment choice for common cold and influenza. *Reference category: Do not use any treatment.*

No.	Variable	Category/ comparison	Herbal remedies		Pharmaceutical treatments		Combined therapy	
			aOR (95% CI)	p-value	aOR (95% CI)	p-value	aOR (95% CI)	p-value
1	Age	Continuous (per year)	1.03 (0.96–1.08)	0.400	0.97 (0.89–1.01)	0.444	0.97 (0.92–1.03)	0.430
2	Sex	Female (Baseline: Male)	**1.87 (1.23–2.86)**	**0.004**	**1.61 (1.01–2.55)**	**0.044**	**2.55 (1.64–3.96)**	**<0.001**
3	Residence	Rural (Baseline: Urban)	0.78 (0.52–1.17)	0.231	**0.57 (0.36–0.89)**	**0.013**	**0.61 (0.40–0.93)**	**0.023**
4	Field of study	Medical (Baseline: non-medical)	1.03 (0.68–1.56)	0.891	0.90 (0.55–1.35)	0.506	0.97 (0.63–1.48)	0.871
5	Presence of chronic diseases	Suffering (Baseline: no chronic disease)	1.12 (0.68–1.84)	0.661	1.23 (0.72–2.10)	0.453	1.49 (0.90–2.47)	0.124
6	Symptoms type	Headache	0.96 (0.63–1.46)	0.854	1.00 (0.63–1.58)	0.958	1.04 (0.67–1.61)	0.859
		Fever	0.69 (0.45–1.04)	0.076	1.07 (0.68–1.68)	0.772	1.03 (0.67–1.58)	0.897
		Sore throat	1.26 (0.82–1.94)	0.297	1.46 (0.91–2.34)	0.120	**1.83 (1.16–2.87)**	**0.009**
		Muscle pain	0.95 (0.62–1.45)	0.797	1.18 (0.75–1.88)	0.476	1.45 (0.93–2.24)	0.100
7	Concern about medication side effects	Possible side effects	**1.60 (1.03–2.49)**	**0.038**	0.90 (0.55–1.44)	0.633	1.06 (0.67–1.68)	0.797
		Long-term dependency	0.81 (0.52–1.26)	0.355	0.78 (0.48–1.27)	0.313	0.82 (0.52–1.30)	0.405
		No concerns	**0.43 (0.24–0.77)**	**0.005**	0.62 (0.33–1.17)	0.137	0.57 (0.31–1.04)	0.064
8	University group	Other universities (Baseline: Yarmouk University)	1.02 (0.68–1.52)	0.938	1.03 (0.68–1.56)	0.880	0.94 (0.64–1.38)	0.758

***Note.*** *aOR, adjusted odds ratio; CI, confidence interval. Bold values indicate statistically significant associations at p < 0.05.*

Overall, 764 participants (42.5%) reported initiating treatment on the same day, 533 (29.7%) after 1–2 days, and 136 (7.6%) after ≥3 days. Regarding first-line treatment choice, 800 participants (44.5%) preferred combined therapy, 476 (26.5%) herbal remedies, 375 (20.9%) pharmaceutical treatments alone, and 146 (8.1%) no treatment.

Sensitivity analysis excluding participants from Yarmouk University showed that the overall pattern of associations was largely preserved (Supplementary S1 Table in S6 File). The institutional grouping variable (other universities vs Yarmouk University) was not significantly associated with first-line treatment choice (all p > 0.05).

Sex was also significantly associated with the distribution of first-line treatment choices (p < 0.001). Combined therapy was reported more frequently by females than males (48.1% vs 36.1%), whereas pharmaceutical treatment alone (19.4% vs 24.3%) and no treatment (6.4% vs 12.2%) were less frequent among females than males.

***Timing of treatment initiation*** – Multinomial logistic regression (Table 3) showed no significant associations between initiation timing and sex, residence, or chronic disease status (all p > 0.05). Medical-field participants had lower odds of initiating treatment on the same day (aOR = 0.55, 95% CI: 0.42–0.72; p < 0.001), while symptom type demonstrated selective effects: headache increased the odds of same-day initiation (aOR = 1.49, 95% CI: 1.14–1.95; p = 0.004), muscle pain increased the odds of initiation after 1–2 days (aOR = 1.64, 95% CI: 1.20–2.24; p = 0.002), and sore throat decreased the odds of same-day initiation (aOR = 0.61, 95% CI: 0.47–0.80; p < 0.001), with a borderline association for initiation after ≥3 days (aOR = 0.67, 95% CI: 0.44–1.02; p = 0.060). Attitudes toward herbal remedies as a complete alternative to pharmaceutical treatments were the strongest predictors, with disagreement and neutral responses generally associated with reduced odds of earlier initiation on the same day or after 1–2 days. Significant associations were also observed for initiation after ≥3 days (disagree: aOR = 0.27, 95% CI: 0.12–0.63; p = 0.003; agree: aOR = 0.57, 95% CI: 0.33–0.99; p = 0.045). The institutional grouping variable (other universities vs Yarmouk University) was not significantly associated with treatment-initiation timing (all p > 0.05).

**Table 3:** Multinomial logistic regression for timing of treatment initiation for common cold or influenza symptoms. *Reference category: Do not use any treatment.*

No.	Variable	Category/ comparison	On the same day		After 1–2 days		After ≥3 days	
			aOR (95% CI)	p-value	aOR (95% CI)	p-value	aOR (95% CI)	p-value
1	Age	Continuous (per year)	0.97 (0.93–1.01)	0.082	0.99 (0.95–1.03)	0.503	0.99 (0.93–1.05)	0.689
2	Sex	Female (Baseline: Male)	1.23 (0.92–1.63)	0.165	1.05 (0.78–1.43)	0.732	0.80 (0.52–1.22)	0.291
3	Residence	Rural (Baseline: Urban)	0.95 (0.73–1.23)	0.682	0.79 (0.60–1.05)	0.105	0.87 (0.57–1.31)	0.498
4	Field of study	Medical (Baseline: non-medical)	**0.55 (0.42–0.72)**	**<0.001**	0.92 (0.70–1.22)	0.552	0.81 (0.54–1.21)	0.301
5	Presence of chronic diseases	Suffering (Baseline: no chronic disease)	1.08 (0.80–1.46)	0.619	0.94 (0.68–1.29)	0.692	1.05 (0.67–1.67)	0.824
6	Symptoms type	Headache	**1.49 (1.14–1.95)**	**0.004**	1.25 (0.94–1.66)	0.123	1.32 (0.87–2.00)	0.195
		Fever	0.96 (0.73–1.25)	0.742	0.91 (0.69–1.21)	0.515	0.99 (0.66–1.49)	0.950
		Muscle pain	1.09 (0.82–1.45)	0.565	**1.64 (1.20–2.24)**	**0.002**	1.01 (0.65–1.56)	0.976
		Sore throat	**0.61 (0.47–0.80)**	**<0.001**	0.88 (0.66–1.17)	0.373	0.67 (0.44–1.02)	0.060
7	I believe that herbal remedies can be a complete alternative to pharmaceutical treatments (Baseline: Strongly Agree)	Strongly Disagree	**0.24 (0.10–0.57)**	**0.001**	**0.07 (0.02–0.33)**	**<0.001**	0.25 (0.05–1.17)	0.078
		Disagree	**0.18 (0.10–0.31)**	**<0.001**	**0.31 (0.18–0.55)**	**<0.001**	**0.27 (0.12–0.63)**	**0.003**
		Neutral	**0.43 (0.30–0.63)**	**<0.001**	**0.55 (0.37–0.81)**	**0.003**	0.59 (0.35–1.01)	0.054
		Agree	0.76 (0.52–1.10)	0.145	0.87 (0.59–1.28)	0.474	**0.57 (0.33–0.99)**	**0.045**
8	University group	Other universities (Baseline: Yarmouk University)	0.83 (0.64–1.08)	0.159	1.16 (0.88–1.53)	0.292	1.35 (0.90–2.03)	0.144

***Note.*** *aOR, adjusted odds ratio; CI, confidence interval. Bold values indicate statistically significant associations at p < 0.05.*

***Response to perceived herbal treatment failure*** – Multinomial logistic regression was performed to examine participants’ self-reported responses when herbal remedies did not achieve the desired results (Table 4), using switching to pharmaceutical treatments as the reference category. Female sex was associated with lower odds of continuing herbal remedies until recovery (aOR = 0.66, 95% CI: 0.49–0.90; p = 0.007), while no significant associations were observed for combining pharmaceutical treatments with herbal remedies or not preferring herbal remedies (p > 0.05). Medical-field participants were also less likely to continue herbal remedies (aOR = 0.72, 95% CI: 0.53–0.98; p = 0.035), with a borderline association for not preferring herbal remedies (aOR = 0.62, 95% CI: 0.37–1.04; p = 0.068). The presence of chronic disease was associated with higher odds of choosing combined therapy rather than switching directly to pharmaceutical treatments (aOR = 1.50, 95% CI: 1.17–1.91; p = 0.001). Reliance on doctor-prescribed pharmaceutical treatments was consistently associated with lower odds of selecting all non-reference responses—continuing herbal remedies (aOR = 0.38, 95% CI: 0.27–0.51; p < 0.001), combining therapy (aOR = 0.56, 95% CI: 0.45–0.70; p < 0.001), and not preferring herbal remedies (aOR = 0.37, 95% CI: 0.23–0.62; p < 0.001)—indicating a greater likelihood of switching directly to pharmaceutical treatments after perceived herbal treatment failure among participants who relied on physician-prescribed treatment.

**Table 4:** Multinomial logistic regression for responses after perceived herbal treatment failure. *Reference category: Switching to pharmaceutical treatments.*

No.	Variable	Category/ comparison	Continue using herbal remedies until recovery		Combine pharmaceutical treatments and herbal remedies		Do not prefer herbal remedies	
			aOR (95% CI)	p-value	aOR (95% CI)	p-value	aOR (95% CI)	p-value
1	Age	Continuous (per year)	1.01 (0.97–1.06)	0.589	1.02 (0.98–1.06)	0.282	1.00 (0.92–1.08)	0.958
2	Sex	Female (Baseline: Male)	**0.66 (0.49–0.90)**	**0.007**	1.03 (0.81–1.32)	0.786	0.73 (0.45–1.20)	0.220
3	Field of study	Medical (Baseline: non-medical)	**0.72 (0.53–0.98)**	**0.035**	0.98 (0.78–1.24)	0.876	0.62 (0.37–1.04)	0.068
4	Presence of chronic diseases	Suffering (Baseline: no chronic disease)	0.86 (0.61–1.21)	0.386	**1.50 (1.17–1.91)**	**0.001**	1.05 (0.60–1.82)	0.871
5	Using pharmaceutical treatments based on the doctor’s prescriptions	Yes (Baseline: No)	**0.38 (0.27–0.51)**	**<0.001**	**0.56 (0.45–0.70)**	**<0.001**	**0.37 (0.23–0.62)**	**<0.001**
6	I believe that herbal remedies could be a complete alternative to pharmaceutical treatments (Baseline: Strongly Agree)	Strongly Disagree	0.90 (0.43–1.87)	0.771	0.66 (0.35–1.20)	0.173	**0.16 (0.03–0.85)**	**0.032**
		Disagree	0.73 (0.39–1.36)	0.332	0.67 (0.43–1.06)	0.086	**0.09 (0.03–0.29)**	**<0.001**
		Neutral	0.67 (0.37–1.21)	0.181	0.82 (0.54–1.24)	0.345	**0.31 (0.14–0.69)**	**0.005**
		Agree	0.61 (0.33–1.11)	0.103	**0.63 (0.41–0.95)**	**0.027**	0.50 (0.24–1.02)	0.056
7	Herbal remedies have a healing ability that equals or exceeds that of pharmaceutical treatments in cases of colds (Baseline: Strongly Agree)	Strongly Disagree	**5.12 (2.10–12.48)**	**<0.001**	0.90 (0.36–2.23)	0.816	Not estimable	—
		Disagree	**2.13 (1.08–4.20)**	**0.030**	1.15 (0.69–1.91)	0.590	1.80 (0.66–4.86)	0.249
		Neutral	1.66 (0.89–3.08)	0.111	1.37 (0.89–2.11)	0.151	0.72 (0.30–1.71)	0.456
		Agree	0.85 (0.45–1.59)	0.603	1.41 (0.94–2.12)	0.096	0.85 (0.41–1.80)	0.675
8	I believe that pharmaceutical treatments are manufactured substances that weaken the immune system, compared to herbal remedies (Baseline: Strongly Agree)	Strongly Disagree	0.80 (0.36–1.79)	0.582	**1.87 (1.01–3.46)**	**0.048**	Not estimable	—
		Disagree	1.31 (0.74–2.30)	0.352	1.22 (0.79–1.87)	0.367	0.41 (0.12–1.38)	0.148
		Neutral	1.13 (0.65–1.94)	0.671	1.33 (0.90–1.95)	0.149	1.75 (0.81–3.75)	0.152
		Agree	1.38 (0.82–2.33)	0.221	1.23 (0.85–1.78)	0.275	1.41 (0.68–2.93)	0.355
9	I believe that herbal remedies have no side effects compared to pharmaceutical treatments (Baseline: Strongly Agree)	Strongly Disagree	**4.49 (2.08–9.71)**	**<0.001**	0.63 (0.27–1.44)	0.271	**5.08 (1.22–21.16)**	**0.026**
		Disagree	1.63 (0.93–2.88)	0.090	0.97 (0.64–1.48)	0.888	1.71 (0.62–4.72)	0.301
		Neutral	**1.88 (1.13–3.12)**	**0.015**	0.87 (0.60–1.26)	0.470	1.33 (0.60–2.96)	0.480
		Agree	1.29 (0.80–2.09)	0.300	0.85 (0.61–1.17)	0.313	0.75 (0.36–1.55)	0.434
10	University group	Other universities (Baseline: Yarmouk University)	1.32 (1.00–1.75)	0.054	**1.46 (1.17–1.82)**	**<0.001**	0.71 (0.42–1.19)	0.195

***Note.*** *aOR, adjusted odds ratio; CI, confidence interval. Bold values indicate statistically significant associations at p < 0.05. “Not estimable” indicates an unstable or unavailable estimate due to sparse data.*

Belief items showed selective associations. For the statement that herbal remedies have a healing ability equal to or exceeding that of pharmaceutical treatments, strong disagreement and disagreement were associated with higher odds of continuing herbal remedies (strongly disagree: aOR = 5.12, 95% CI: 2.10–12.48; p < 0.001; disagree: aOR = 2.13, 95% CI: 1.08–4.20; p = 0.030). Strong disagreement with the statement that pharmaceutical treatments weaken the immune system compared with herbal remedies was associated with higher odds of choosing combined therapy (aOR = 1.87, 95% CI: 1.01–3.46; p = 0.048). Finally, for the belief that herbal remedies have no side effects compared with pharmaceutical treatments, strong disagreement and neutral responses were associated with higher odds of continuing herbal remedies (strongly disagree: aOR = 4.49, 95% CI: 2.08–9.71; p < 0.001; neutral: aOR = 1.88, 95% CI: 1.13–3.12; p = 0.015), while strong disagreement was also associated with higher odds of not preferring herbal remedies (aOR = 5.08, 95% CI: 1.22–21.16; p = 0.026).

Institutional grouping showed an outcome-specific association: participants from universities other than Yarmouk University had higher odds of combining pharmaceutical treatments with herbal remedies rather than switching directly to pharmaceutical treatments (aOR = 1.46, 95% CI: 1.17–1.82; p < 0.001), whereas the other comparisons were not statistically significant.

***Medication-obtaining behavior*** – A multinomial logistic regression model was used to examine medication-obtaining behavior (buying pharmaceutical treatments without a prescription based on personal experience, consulting only a pharmacist, or not preferring pharmaceutical treatments), using doctor consultation and prescription-based use as the reference category (Table 5). Field of study was a significant predictor; participants in the medical field had higher odds of buying pharmaceutical treatments without a prescription based on their own experience (aOR = 1.76, 95% CI: 1.39–2.23; p < 0.001) and of not preferring pharmaceutical treatment use (aOR = 1.84, 95% CI: 1.27–2.66; p = 0.001). Residence was also significant for the “consult only a pharmacist” category, with rural participants having lower odds than urban participants (aOR = 0.64, 95% CI: 0.49–0.83; p < 0.001). Regarding symptom type, reporting muscle pain was associated with higher odds of buying pharmaceutical treatments without a prescription (aOR = 1.31, 95% CI: 1.00–1.70; p = 0.049). No significant associations were observed for age, sex, chronic disease status, or the other symptom categories (all remaining p > 0.05).

**Table 5:** Multinomial logistic regression for medication-obtaining behavior. *Reference category: Doctor consultation and prescription-based use.*

No.	Variable	Category/ comparison	Buy pharmaceutical treatments without a prescription based on own experience		Consult only a pharmacist		Do not prefer pharmaceutical treatments	
			aOR (95% CI)	p-value	aOR (95% CI)	p-value	aOR (95% CI)	p-value
1	Age	Continuous (per year)	1.04 (1.00–1.08)	0.061	1.01 (0.97–1.05)	0.719	1.04 (0.99–1.11)	0.133
2	Sex	Female (Baseline: Male)	0.95 (0.74–1.22)	0.673	1.25 (0.95–1.66)	0.113	0.81 (0.55–1.20)	0.295
3	Residence	Rural (Baseline: Urban)	0.89 (0.70–1.12)	0.313	**0.64 (0.49–0.83)**	**<0.001**	0.94 (0.65–1.36)	0.739
4	Field of study	Medical (Baseline: non-medical)	**1.76 (1.39–2.23)**	**<0.001**	1.22 (0.95–1.57)	0.114	**1.84 (1.27–2.66)**	**0.001**
5	Presence of chronic diseases	Suffering (Baseline: no chronic disease)	0.99 (0.76–1.28)	0.909	1.14 (0.86–1.50)	0.363	0.75 (0.48–1.17)	0.204
6	Symptoms type	Headache	0.99 (0.73–1.26)	0.910	0.90 (0.70–1.17)	0.435	0.76 (0.53–1.10)	0.149
		Fever	0.95 (0.75–1.21)	0.673	1.05 (0.81–1.35)	0.716	0.99 (0.68–1.43)	0.936
		Muscle pain	**1.31 (1.00–1.70)**	**0.049**	1.06 (0.80–1.39)	0.692	1.06 (0.71–1.60)	0.762
		Sore throat	1.05 (0.82–1.34)	0.706	1.06 (0.82–1.38)	0.649	1.22 (0.84–1.77)	0.305
7	University group	Other universities (Baseline: Yarmouk University)	**1.50 (1.18–1.91)**	**<0.001**	**1.53 (1.19–1.98)**	**<0.001**	1.28 (0.88–1.86)	0.201

***Note.*** *aOR, adjusted odds ratio; CI, confidence interval. Bold values indicate statistically significant associations at p < 0.05.*

Institutional grouping was also associated with selected medication-obtaining behaviors. Compared with Yarmouk University participants, students from other universities had higher odds of purchasing pharmaceutical treatments without a prescription based on personal experience (aOR = 1.50, 95% CI: 1.18–1.91; p < 0.001) and consulting only a pharmacist (aOR = 1.53, 95% CI: 1.19–1.98; p < 0.001), whereas no significant association was observed for not preferring pharmaceutical treatments (p = 0.201).

Additionally, among the 1,797 participants, 867 (48.2%) self-reported antibiotic use for common cold or influenza symptoms. This proportion represents overall self-reported antibiotic use, regardless of how antibiotics were obtained, including through medical prescription, previous personal experience, or pharmacist advice. Because antibiotic type, indication, dose, duration, and clinical appropriateness were not collected, this finding should be interpreted as reported antibiotic use rather than confirmed inappropriate antibiotic use. Among herbal remedies, sage (54.3%), chamomile (43.9%), anise (40.1%), and ginger (37.5%) were the most commonly preferred choices among participants.

## Discussion

***Interpretation of Main Findings*** – The main finding of this study was the predominance of mixed or integrative self-care, with combined use of herbal remedies and pharmaceutical treatments emerging as the most common first-line choice for common cold and influenza symptoms. This finding suggests that many Jordanian university students do not view herbal remedies and pharmaceutical treatments as mutually exclusive options, but rather combine them as part of their self-management practices. This pattern is consistent with the regional cultural context, where herbal remedies remain familiar and socially accepted, while pharmaceutical treatments are also widely accessible and perceived as effective for symptom relief.

Sex was an important predictor of first-line treatment choice. Female participants had higher odds of choosing all active treatment options compared with males, particularly combined use of herbal remedies and pharmaceutical treatments (aOR = 2.55, 95% CI: 1.64–3.96; p < 0.001). This pattern is consistent with previous evidence suggesting that women are generally more proactive in health-seeking behavior and more likely to use medications [21,22]. In the present study, this may reflect greater willingness among female students to use multiple symptom-relief strategies rather than relying on no treatment.

Field of study was most relevant to medication-obtaining behavior. Medical-field participants had higher odds of purchasing pharmaceutical treatments without a prescription, which may reflect greater confidence in self-management and familiarity with treatment options among students with health-related academic exposure [23]. However, this finding should not be interpreted as evidence of appropriate medication use, because the study assessed self-reported behavior rather than clinical appropriateness. The association between muscle pain and non-prescription pharmaceutical treatment purchase is also plausible, as analgesics are commonly used in self-medication among university students [24].

Timing of treatment initiation was associated with selected symptoms and beliefs. Participants reporting headache were more likely to initiate treatment on the same day, whereas those reporting sore throat were less likely to do so. These findings suggest that treatment timing may depend not only on symptom presence but also on how students perceive symptom severity and the need for immediate relief. The association between belief in herbal remedies as a complete alternative and treatment initiation further supports the role of health beliefs in shaping self-care behavior.

***Regional and Population-Specific Considerations*** – The regional context is important when interpreting these findings. Jordan is located in the Middle East, a region with a long tradition of herbal medicine use and rich medicinal plant diversity, with more than 2,600 plant species reported in the region and several still used in Arab traditional medicine [17]. In this context, the continued use of herbal remedies for common cold and influenza symptoms is not unexpected. Rather, it likely reflects cultural familiarity, perceived safety, household availability, and intergenerational advice. At the same time, the frequent use of pharmaceutical treatments and combined therapy suggests that students often integrate traditional and modern treatment approaches rather than relying exclusively on one modality.

Residence was also relevant. Rural participants showed lower odds of pharmaceutical and combined treatment use than urban participants, while residence was not significantly associated with choosing herbal remedies alone. This may reflect differences in access, treatment availability, health-seeking norms, or preferences for specific sources of advice between rural and urban settings. However, because this study did not directly assess healthcare access, pharmacy density, or household treatment practices, these explanations should be interpreted cautiously.

### *Comparison with Previous Literature* –

1. First-line treatment choice and treatment initiation: In this study, the combined use of herbal remedies and pharmaceutical treatments was the most frequently selected first-line treatment approach for common cold and influenza symptoms. This finding is consistent with regional evidence showing the continued use of herbal remedies for respiratory and self-limiting symptoms, including findings from Western Saudi Arabia, where herbal remedy use was commonly reported [25]. Although that study did not assess combined therapy as a separate category, both findings suggest that herbal remedies remain an important component of symptom management in the region. The high proportion of students initiating treatment on the same day or within 1–2 days of symptom onset (72.2%) also suggests early self-management behavior. This proportion is higher than that reported in a United States study, in which 35.0% of adults sought care within two days of influenza-like illness onset [26]. However, direct comparison should be made cautiously because that study assessed healthcare-seeking rather than self-treatment initiation.
2. Response to perceived herbal treatment failure: Medical-field participants were less likely to continue herbal remedies after perceived herbal treatment failure. This may reflect greater exposure to biomedical training and evidence-based treatment concepts. Previous research suggests that healthcare professionals may have concerns regarding the safety monitoring and effectiveness of herbal medicine [27]. In addition, prior work has shown that perceptions of complementary and alternative medicine may vary by training level and exposure [28]. The association between chronic disease and choosing combined therapy rather than switching directly to pharmaceutical treatment may reflect a tendency among individuals with chronic conditions to use complementary approaches alongside conventional treatment, as reported in previous studies of chronic disease populations [29,30].
3. Medication-obtaining behavior: Medical-field participants had higher odds of purchasing pharmaceutical treatments without a prescription compared with physician-based prescription users. This is consistent with previous evidence indicating that self-medication and self-prescription behaviors may be common among medical students [23]. Rural participants had lower odds of relying exclusively on pharmacists than urban participants. This finding differs from some studies reporting higher pharmacy-based medication use or self-medication in rural settings [31,32], which may reflect differences in healthcare access, study populations, healthcare systems, and definitions of self-medication. Finally, muscle pain was associated with higher odds of purchasing pharmaceutical treatments without a prescription, which is plausible given that analgesics are commonly used in self-medication practices among university students [24].

***Strengths and Limitations –***This study addresses an important gap in the limited local evidence from Jordan regarding treatment preferences for common cold and influenza symptoms among university students. In addition to examining overall treatment preference, it assessed multiple dimensions of treatment-related behavior, including first-line treatment choice, timing of treatment initiation, responses to perceived herbal treatment failure, and medication-obtaining behavior, thereby providing a more comprehensive view of students’ self-management practices. Another strength of this study is its large final sample (n = 1,797), which exceeded the minimum sample size estimated a priori through power analysis (n = 1,628), supporting adequate statistical power and more precise estimates. Moreover, the use of multinomial logistic regression enabled simultaneous comparisons across multiple treatment-choice categories while adjusting for potential confounders, allowing for a more nuanced evaluation of treatment behaviors. The analysis also incorporated a broad range of covariates, including demographic characteristics, symptoms, and belief- and awareness-related factors, which strengthened the assessment of independent associations. In addition, the robustness of the first-line treatment-choice findings was supported by a sensitivity analysis excluding participants from Yarmouk University, in which the overall pattern of associations in that model remained largely unchanged. Finally, the study was reported in accordance with the STROBE guidelines for cross-sectional studies [20], which enhances transparency and reproducibility.

This study also has several limitations. First, participants from Yarmouk University were overrepresented in the sample (63.3%), reflecting the convenience sampling approach and potentially limiting the generalizability of findings to all Jordanian university students. To address this issue, we performed a sensitivity analysis excluding Yarmouk University respondents and adjusted for university group in the regression models. Although university group showed some outcome-specific associations, the main findings were not materially altered. Second, the cross-sectional design precludes causal inference, and the observed relationships should therefore be interpreted as associations rather than causal effects. Third, the study relied on self-reported data, which may be subject to recall bias, reporting bias, and social desirability bias. In addition, Questions 12–15 were phrased in the present tense and did not explicitly distinguish between participants’ usual practices, previous experiences, and hypothetical responses. Consequently, participants may have interpreted these items differently, introducing potential measurement error. In addition, common cold and influenza were assessed as self-reported symptom-based conditions rather than clinically or laboratory-confirmed diagnoses, which may have led to misclassification of illness type. The online survey format may also have influenced responses. Medication-obtaining behavior and several multiple-response questionnaire items were assessed using predefined closed-ended categories without a free-text “Other” option. Consequently, the questionnaire may not have captured less common herbal remedies, pharmaceutical treatments, motivations, concerns, information sources, or alternative sources of treatment advice and access, such as family members, friends, or online information. The study also did not assess the dose, frequency, duration, preparation method, or concurrent timing of herbal and pharmaceutical treatment use, limiting the ability to evaluate safety, interactions, or appropriateness of combined therapy. Fourth, although an economic-status item was included in the questionnaire, it did not adequately capture family economic status because many participants appeared to interpret it as personal student economic status, and responses were highly concentrated in one category. Therefore, family economic status was not included in the analysis, and its potential influence on treatment preference and medication-obtaining behavior could not be assessed. Fifth, the study assessed treatment preferences, beliefs, and self-reported behaviors, but did not directly measure participants’ objective knowledge about common cold, influenza, herbal remedies, pharmaceutical treatments, or antibiotic use. Sixth, although antibiotic use was assessed, detailed information on antibiotic type, indication, dose, duration, and whether use was clinically appropriate was not collected. Therefore, antibiotic-related findings should be interpreted as self-reported antibiotic use rather than confirmed inappropriate antibiotic use. Finally, although the questionnaire underwent expert review and pilot testing, it was developed for this study and did not undergo full external psychometric validation.

***Clinical and Public Health Implications*** – The present findings have important clinical and public health implications. First, the frequent reliance on self-directed treatment, particularly combined therapy, indicates a need for targeted health education among university students. Second, the observed influence of beliefs about treatment effectiveness and safety suggests that clinicians should address misconceptions and provide culturally sensitive counseling on the appropriate use of herbal and pharmaceutical treatments. Third, the frequent use of over-the-counter pharmaceutical treatments and the notable level of self-reported antibiotic use highlight the need to strengthen rational medication-use education and antibiotic stewardship for viral respiratory illnesses. In practice, these findings also support closer collaboration between physicians and pharmacists to guide safe self-care and identify inappropriate medication use. Overall, the findings support targeted educational strategies to improve health literacy and reduce inappropriate self-medication among young adults.

***Future Research –*** This study highlights several avenues for future research. Given the observed association between beliefs and treatment-related behaviors, future studies should explore in greater depth the underlying psychological and cultural factors that shape these patterns, ideally using validated measurement tools and longitudinal designs. Qualitative research is also recommended to better understand the motivations behind early treatment initiation and the preference for combined therapy.

## Conclusion

This study suggests that Jordanian university students commonly use integrative self-management approaches for cold and flu symptoms, with combined use of herbal remedies and pharmaceutical treatments as the most frequent pattern. Treatment preferences were associated with demographic factors, particularly sex and place of residence, while concerns about medication side effects were linked to greater preference for herbal remedies among some students. Medical-field students had lower adjusted odds of continuing herbal remedies after perceived herbal treatment failure, relative to switching to pharmaceutical treatments, suggesting that academic background may influence treatment-related decisions. The notable level of self-reported antibiotic use further highlights the need for stronger education on appropriate self-management, rational medication use, and antibiotic stewardship for common viral respiratory illnesses.

## Supporting information

S1 File STROBE checklist. Completed STROBE checklist for cross-sectional studies.
(PDF)

S2 File Original Arabic questionnaire as administered. (PDF)

S3 File English translation of the original questionnaire as administered. (PDF)

S4 File Post-study revised English questionnaire. Revised version prepared after completion of data collection for potential future use; not administered in the present study.
(PDF)

S5 File Sample size calculation. G*Power output for the a priori sample size calculation.
(PDF)

S6 File Supplementary tables. Table S1 presents the sensitivity analysis excluding participants from Yarmouk University; Table S2 presents the pattern matrix for the refined 8-item belief scale; Table S3 presents descriptive statistics for all independent variables included in the multinomial logistic regression models; Table S4 presents the association between sex and first-line treatment choice; and Table S5 presents the exploratory factor analysis and internal consistency results for the refined 8-item belief scale.
(PDF)

S7 File Anonymized minimal dataset. (XLSX)

## References

[1] World Health Organization. Trends of acute respiratory infection, including human metapneumovirus, in the Northern Hemisphere. https://www.who.int/emergencies/disease-outbreak-news/item/2025-DON550. 2025.

[2] World Health Organization. WHO updates influenza care guidelines: includes recommendations for viruses with pandemic potential. https://www.who.int/news/item/13-09-2024-who-updates-influenza-care-guidelines--includes-recommendations-for-viruses-with-pandemic-potential. 2024.

[3] World Health Organization. Influenza [Internet]. 2009. Available from: https://www.who.int/europe/news-room/fact-sheets/item/influenza

[4] Allan GM, Arroll B. Prevention and treatment of the common cold: making sense of the evidence. CMAJ. 2014;186(3):190–9. doi: 10.1503/cmaj.121442 24468694 PMC3928210

[5] Krammer F, Smith GJD, Fouchier RAM, Peiris M, Kedzierska K, Doherty PC. Influenza. Nat Rev Dis Primers. 2018;4(1):3. doi: 10.1038/s41572-018-0002-y 29955068 PMC7097467

[6] Romanelli RJ, Cabling M, Marciniak-Nuqui Z, Marjanovic S, Morris S, Dufresne E, et al. The Societal and Indirect Economic Burden of Seasonal Influenza in the United Kingdom. Rand Health Q. 2023;10(4):2. doi: 10.7249/rhq-10-04-02 37720072 PMC10501821

[7] Bartkowiak V. The common cold and influenza conundrum: getting it right. IQVIA. https://www.iqvia.com/blogs/2018/02/the-cold-and-flu-conundrum-getting-it-right. 2018. Accessed 2026 February 27.

[8] World Health Organization. Traditional medicine. https://www.who.int/news-room/questions-and-answers/item/traditional-medicine. 2023. Accessed 2023.

[9] Geck MS, Cristians S, Berger-González M, Casu L, Heinrich M, Leonti M. Traditional Herbal Medicine in Mesoamerica: Toward Its Evidence Base for Improving Universal Health Coverage. Front Pharmacol. 2020;11:1160. doi: 10.3389/fphar.2020.01160 32848768 PMC7411306

[10] World Health Organization. WHO global report on traditional and complementary medicine 2019. Geneva: World Health Organization. 2019. https://iris.who.int/handle/10665/312342

[11] Kartiani A. Cultural and Socioeconomic Influences on Medicine Choice: A Comparative Review of Herbal and Pharmaceutical Drug Preferences and Their Implications for Health Policy. jrkpk. 2022;1(2):72–86. doi: 10.61194/jrkpk.v1i2.676

[12] Tian Y-S, Mao X, Zhou Y, Fukuzawa K, Ikeda K, Hatabu A. Status and influencing factors of OTC medicine use for self-medication in cold and cough: a cross-sectional survey in Japan. BMC Public Health. 2025;25(1):1918. doi: 10.1186/s12889-025-23113-4 40413411 PMC12102812

[13] Malak MZ, AbuKamel AM. Self-medication practices among university students in Jordan. Malays J Med Health Sci. 2019;15(2):112–9.

[14] Gutema GB, Gadisa DA, Kidanemariam ZA, Berhe DF, Berhe AH, Hadera MG. Self-medication practices among health sciences students: the case of Mekelle University. J Appl Pharm Sci. 2011;1(10):183–9.

[15] Azhar MIM, Gunasekaran K, Kadirvelu A, Gurtu S, Sadasivan S, Kshatriya BM. Self-medication: awareness and attitude among Malaysian urban population. Int J Collab Res Intern Med Public Health. 2013;5(6):436–43.

[16] Alshogran OY, Alzoubi KH, Khabour OF, Farah S. Patterns of self-medication among medical and nonmedical University students in Jordan. Risk Manag Healthc Policy. 2018;11:169–76. doi: 10.2147/RMHP.S170181 30254501 PMC6143637

[17] Saad B, Azaizeh H, Said O. Tradition and perspectives of arab herbal medicine: a review. Evid Based Complement Alternat Med. 2005;2(4):475–9. doi: 10.1093/ecam/neh133 16322804 PMC1297506

[18] World Health Organization. Antimicrobial resistance. https://www.who.int/news-room/fact-sheets/detail/antimicrobial-resistance. 2023.

[19] Centers for Disease Control and Prevention. Healthy habits: antibiotic do’s and don’ts. https://www.cdc.gov/antibiotic-use/about/index.html. 2025.

[20] von Elm E, Altman DG, Egger M, Pocock SJ, Gøtzsche PC, Vandenbroucke JP, et al. The Strengthening the Reporting of Observational Studies in Epidemiology (STROBE) statement: guidelines for reporting observational studies. PLoS Med. 2007;4(10):e296. doi: 10.1371/journal.pmed.0040296 17941714 PMC2020495

[21] Orlando V, Mucherino S, Guarino I, Guerriero F, Trama U, Menditto E. Gender Differences in Medication Use: A Drug Utilization Study Based on Real World Data. Int J Environ Res Public Health. 2020;17(11):3926. doi: 10.3390/ijerph17113926 32492925 PMC7312791

[22] Manteuffel M, Williams S, Chen W, Verbrugge RR, Pittman DG, Steinkellner A. Influence of patient sex and gender on medication use, adherence, and prescribing alignment with guidelines. J Womens Health (Larchmt). 2014;23(2):112–9. doi: 10.1089/jwh.2012.3972 24206025

[23] Sawalha K, Sawalha A, Salih E, Aldhuhori N, Aboukalam N, Bakie R. Health seeking behavior among medical students in the University of Sharjah. J Pharm Pharmacol. 2017;5(8):561–4. doi: 10.17265/2328-2150/2017.08.011

[24] Chindhalore CA, Dakhale GN, Giradkar AB. Comparison of self-medication practices with analgesics among undergraduate medical and paramedical students of a tertiary care teaching institute in Central India - A questionnaire-based study. J Educ Health Promot. 2020;9:309. doi: 10.4103/jehp.jehp_378_20 33426113 PMC7774615

[25] Zaidi SF, Saeed SA, Khan MA, Khan A, Hazazi Y, Otayn M, et al. Public knowledge, attitudes, and practices towards herbal medicines; a cross-sectional study in Western Saudi Arabia. BMC Complement Med Ther. 2022;22(1):326. doi: 10.1186/s12906-022-03783-y 36482398 PMC9733054

[26] Biggerstaff M, Jhung MA, Reed C, Fry AM, Balluz L, Finelli L. Influenza-like illness, the time to seek healthcare, and influenza antiviral receipt during the 2010-2011 influenza season-United States. J Infect Dis. 2014;210(4):535–44. doi: 10.1093/infdis/jiu224 24731959 PMC4610008

[27] Hasen G, Hashim R. Current Awareness of Health Professionals on the Safety of Herbal Medicine and Associated Factors in the South West of Ethiopia. J Multidiscip Healthc. 2021;14:2001–8. doi: 10.2147/JMDH.S321765 34349517 PMC8326526

[28] Kessler RC, Davis RB, Foster DF, Van Rompay MI, Walters EE, Wilkey SA, et al. Long-term trends in the use of complementary and alternative medical therapies in the United States. Ann Intern Med. 2001;135(4):262–8. doi: 10.7326/0003-4819-135-4-200108210-00011 11511141

[29] Caballero-Hernández CI, González-Chávez SA, Urenda-Quezada A, Reyes-Cordero GC, Peláez-Ballestas I, Álvarez-Hernández E, et al. Prevalence of complementary and alternative medicine despite limited perceived efficacy in patients with rheumatic diseases in Mexico: Cross-sectional study. PLoS One. 2021;16(9):e0257319. doi: 10.1371/journal.pone.0257319 34582473 PMC8478211

[30] Thilakarathna M, Appuhami K, Darshana N, Perera J. Complementary and alternative medicine use among patients with type-2 diabetes mellitus attending a suburban tertiary healthcare centre in Sri Lanka. BMC Complement Med Ther. 2025;25(1):363. doi: 10.1186/s12906-025-05077-5 41068695 PMC12512381

[31] Supriya CK, Prakash C, Nandini T, Reddy PPK. A comparative study on the knowledge, attitudes, and practices of self-medication in rural and urban regions of Tumkur, Karnataka, India. MGM J Med Sci. 2025;12(2):240–8. doi: 10.4103/mgmj.mgmj_92_25

[32] Ge J, Sun X, Meng H, Risal PG, Liu D. Factors associated with self-medication in children and the decomposition of rural-urban disparities in China. BMC Public Health. 2021;21(1):2123. doi: 10.1186/s12889-021-12137-1 34794400 PMC8603473

