# Supplementary material for: Herbal or conventional medicine? A cross-sectional study of Jordanian university students’ preferences for cold and flu treatment
Source: PLoS One. 2026 Sep 15;21(9):e0358179. doi: 10.1371/journal.pone.0358179 (PMC13577359; doi:10.1371/journal.pone.0358179)
Supplement: Supplementary file 2 [file pone.0358179.s002.pdf]

علاج نزلات البرد والإنفلونزا: الأعشاب أم الأدوية؟

تهدف هذه الدراسة إلى فهم تفضيلات طلاب الجامعات في اختيار علاجات نزلات البرد والإنفلونزا بين استخدام الأعشاب والطب التقليدي، وذلك من خلال استبيان يسلط الضوء على العوامل المؤثرة في اتخاذ القرار، مثل الفعالية المتصورة، والأمان، والتجارب السابقة. تسعى النتائج إلى توفير معلومات قيمة لمقدمي الرعاية الصحية حول توجهات المرضى، بما يساهم في تحسين التوعية وتقديم خيارات علاجية تتماشى مع احتياجات المجتمع.

- يتطلب ملء هذا الاستبيان حوالي 5 دقائق.
- إجاباتك مهمة لدعم البحث العلمي وتطوير المعرفة الطبية.
- جميع المعلومات التي ستتم مشاركتها ستُستخدم فقط لأغراض البحث وبخصوصية تامة، ولا توجد معلومات شخصية مطلوبة من المشاركين.

الموافقة على المشاركة

1. ملء هذا الاستبيان يُعتبر تطوعياً، وستُؤخذ الردود بصيغة مجهولة للمحافظة على خصوصية المشاركين. هل توافق على المشاركة في هذا الاستبيان؟

○ أوافق

المعلومات الشخصية / الديموغرافية

2. الجنس

○ ذكر ○ أنثى

3. العمر

اختر إجابة واحدة.

○ 18 ○ 19 ○ 20 ○ 21 ○ 22 ○ 23
○ 24 ○ 25 ○ 26 ○ 27 ○ 28 ○ 29
○ 30 ○ 31 ○ 32 ○ 33 ○ 34 ○ 35

4. مكان الإقامة

○ منطقة حضرية ○ منطقة ريفية

المعلومات الشخصية / الديموغرافية - تابع

5. الحالة الاجتماعية

○ أعزب ○ متزوج/ة ○ مطلق/ة أو أرمل/ة

6. الدخل بالدينار الأردني

○ 500-0 ○ 1000-500
○ 2000-1000 ○ أكثر من 2000

7. الجامعة

○ جامعة اليرموك
○ الجامعة الهاشمية
○ جامعة مؤتة
○ جامعة الحسين بن طلال
○ الجامعة الألمانية الأردنية
○ جامعة خاصة
○ جامعة العلوم والتكنولوجيا الأردنية
○ الجامعة الأردنية
○ جامعة البلقاء التطبيقية
○ جامعة آل البيت
○ جامعة الطفيلة التقنية
○ جامعة الحسين التقنية

8. التخصص

○ طبي ○ غير طبي

9. السنة الدراسية

- ○ الأولى
- ○ الثانية
- ○ الثالثة
- ○ الرابعة
- ○ الخامسة
- ○ السادسة
- ○ خريج/ة

10. التقدير الدراسي

- ○ ممتاز
- ○ جيد جداً
- ○ جيد
- ○ مقبول

11. هل تعاني من أي أمراض مزمنة؟

- ○ نعم
- ○ لا

اتجاهات واختيارات الأفراد في التعامل مع نزلات البرد والإنفلونزا

12. ما الأعراض المرضية التي تشعر بها عند إصابتك بنزلة برد وتشعر أنك بحاجة إلى علاج؟

يمكن اختيار أكثر من خيار.

- ☐ صداع
- ☐ حمى
- ☐ آلام عضلية
- ☐ آلام في الحلق

13. إذا كنت تفضل استخدام العلاجات عند الإصابة بنزلة برد أو إنفلونزا، متى تبدأ باستخدام العلاج بعد ظهور الأعراض؟

- ○ في نفس اليوم
- ○ بعد يوم إلى يومين
- ○ لا أفضل استخدام العلاج
- ○ بعد 3 أيام أو أكثر

14. عند إصابتك بنزلة برد أو إنفلونزا، ما خيارك الأول للعلاج؟

- ○ الأعشاب الطبيعية (مثل الزنجبيل، البابونج، الزعتر، الميرمية)
- ○ استخدام الأدوية (مثل البنادول، مضادات الاحتقان، المضادات الحيوية عند الحاجة)
- ○ الجمع بين الاثنين
- ○ لا أفضل أي علاج وسأعتمد على الراحة

15. ما السبب الرئيسي لاختيارك هذا النوع من العلاج؟

يمكن اختيار أكثر من خيار.

- ☐ فعالية أسرع
- ☐ آثار جانبية أقل
- ☐ تكلفة أقل
- ☐ تجربة شخصية ناجحة
- ☐ متاح بسهولة

استخدام العلاجات العشبية

16. هل تعتمد على العلاجات العشبية عند المرض؟

- ○ نعم
- ○ لا

17. إذا كنت تفضل استخدام العلاجات العشبية (الأعشاب) عند إصابتك بنزلة برد، متى تبدأ باستخدام العلاج بعد ظهور الأعراض؟

- ○ في نفس اليوم
- ○ بعد يوم إلى يومين
- ○ أنا لا أفضل استخدام الأعشاب
- ○ بعد 3 أيام أو أكثر

18. ما الأعشاب التي تستخدمها عادة عند الإصابة بنزلة برد أو إنفلونزا؟

يمكن اختيار أكثر من خيار.

- ☐ زنجبيل
- ☐ بابونج
- ☐ زعتر
- ☐ ميرمية
- ☐ يانسون
- ☐ كركم
- ☐ نعنع
- ☐ أنا لا أفضل استخدام الأعشاب

19. ما الدافع لاستخدام الأعشاب في حالات نزلات البرد؟

يمكن اختيار أكثر من خيار.

- ☐ عدم توفر الوقت لزيارة الطبيب
- ☐ التكلفة العالية لمراجعة الطبيب
- ☐ موصى بها من قبل الأصدقاء أو الأسرة
- ☐ تجربة سابقة
- ☐ المسافة البعيدة للعيادات أو المستشفيات عن المنزل
- ☐ أعاني من أعراض بسيطة وسهلة العلاج
- ☐ موصى بها من قبل الصيدلي
- ☐ الإعلانات
- ☐ لا أفضل استخدام الأعشاب

20. إذا لم تحقق الأعشاب الطبيعية النتائج المرجوة، هل ستلجأ إلى العلاج بالأدوية؟

- ○ نعم، سأتجه إلى الأدوية
- ○ قد أدمج بين الأعشاب والأدوية
- ○ لا، سأستمر في استخدام الأعشاب حتى أتعافى
- ○ أنا لا أفضل استخدام الأعشاب

استخدام الأدوية الصيدلانية

21. إذا كنت تفضل استخدام العلاجات عند المرض، متى تبدأ باستخدام العلاج بعد ظهور الأعراض؟

- ○ في نفس اليوم
- ○ بعد 3 أيام أو أكثر
- ○ بعد يوم إلى يومين
- ○ لا أفضل استخدام العلاجات

22. في حال اللجوء إلى الأدوية، هل تشتريها بناءً على وصفة طبية؟

- ○ نعم، أنا أستشير الطبيب دائماً قبل شراء أي دواء
- ○ لا، أنا أشتري الأدوية بدون وصفة طبية بناءً على تجربتي ومعرفتي
- ○ أستشير الصيدلي فقط
- ○ أنا لا أفضل استخدام الأدوية

23. ما الأدوية التي تستخدمها عادة عند الإصابة بنزلة برد أو إنفلونزا؟

يمكن اختيار أكثر من خيار.

- ☐ المسكنات (مثل البنادول)
- ☐ مضادات الهيستامين
- ☐ أنا لا أفضل استخدام الأدوية
- ☐ المضادات الحيوية (مثل أموكلان)
- ☐ مزيلات الاحتقان (مثل السودوإيفيدرين)

24. ما العوامل التي تؤثر على اختيارك بين الأدوية الصيدلانية؟

يمكن اختيار أكثر من إجابة.

- ☐ شدة الأعراض
- ☐ توصيات الأطباء
- ☐ أنا لا أفضل استخدام الأدوية
- ☐ توفر العلاج وسهولة الحصول عليه
- ☐ قلة الآثار الجانبية

25. ما أكثر ما يقلقك بشأن استخدام الأدوية الطبية؟

يمكن اختيار أكثر من إجابة.

- ☐ الآثار الجانبية المحتملة
- ☐ مقاومة الجسم للأدوية
- ☐ لا يوجد أي قلق بشأن استخدامها
- ☐ التكلفة العالية
- ☐ الاعتماد عليها على المدى الطويل

المعتقدات والاتجاهات نحو العلاجات العشبية والأدوية

26. يرجى تحديد مدى موافقتك أو عدم موافقتك على كل عبارة.

اختر إجابة واحدة في كل صف.

العبارة	موافق بشدة	موافق	محايد	غير موافق	غير موافق بشدة
1. أعتقد أن تجربتي الشخصية في استخدام الأعشاب / الأدوية مرتبطة بتجارب بعض الناس من حولي (الأهل، الأصدقاء، المجتمع).	○	○	○	○	○
2. استخدام بعض الأعشاب الطبية كمكون أساسي في تصنيع بعض الأدوية يسهم في تشكيل توجهات المجتمع نحو الطب الحديث.	○	○	○	○	○
3. أتخلى عن دواء معين لعلاج نزلات البرد عند سماعي عن أعشاب طبية تعالجها.	○	○	○	○	○
4. إذا نصح الطبيب باستخدام الأعشاب سأكون أكثر ميلاً لتجربتها.	○	○	○	○	○
5. أعتقد أن الأعشاب قد تكون بديلاً كاملاً عن الأدوية.	○	○	○	○	○
6. أعتقد أن الأدوية أكثر فعالية من العلاجات العشبية.	○	○	○	○	○
7. الأعشاب لديها قدرة علاجية تعادل أو تفوق الأدوية في حالات نزلات البرد.	○	○	○	○	○
8. أؤمن بأن الأدوية مواد مصنعة تضعف المناعة مقارنة بالعلاجات العشبية.	○	○	○	○	○
9. أؤمن بأن الأعشاب ليس لها أي آثار جانبية مقارنة بالأدوية.	○	○	○	○	○
10. أعتقد أن بعض العلاجات العشبية قد تسبب تفاعلات تحسسية، لذا يجب استخدامها بحذر وبعد التأكد من ملاءمتها الشخصية.	○	○	○	○	○

مصادر المعلومات

27. من أين تحصل على معلوماتك حول فعالية الأعشاب والأدوية في علاج نزلات البرد والإنفلونزا؟

يمكن اختيار أكثر من خيار.

☐ مصادر أكاديمية / كتب علمية
☐ الأطباء والصيادلة
☐ الإنترنت ووسائل التواصل الاجتماعي
☐ تجارب شخصية أو توصيات الأهل والأصدقاء

شكراً لمشاركتك

نقدّر وقتك ومساهمتك في دعم البحث العلمي.
