# Supplementary material for: Herbal or conventional medicine? A cross-sectional study of Jordanian university students’ preferences for cold and flu treatment
Source: PLoS One. 2026 Sep 15;21(9):e0358179. doi: 10.1371/journal.pone.0358179 (PMC13577359; doi:10.1371/journal.pone.0358179)
Supplement: Supplementary file 3 [file pone.0358179.s003.pdf]

ENGLISH QUESTIONNAIRE

Treatment of Common Cold and Influenza

Herbal Remedies or Medications?

Faithful English translation of the supplied Arabic questionnaire

Study purpose

This study aims to understand university students' preferences when choosing treatments for common cold and influenza, including herbal remedies and conventional medications. The questionnaire highlights factors that may influence treatment decisions, such as perceived effectiveness, safety, and previous experiences. The findings are intended to provide healthcare professionals with valuable information about patients' preferences, thereby supporting improved health education and treatment options that align with community needs.

- Completing this questionnaire takes approximately 5 minutes.
- Your responses are important for supporting scientific research and advancing medical knowledge.
- All information will be used solely for research purposes and treated with strict confidentiality. No identifying personal information is required.

Consent

1 **Completing this questionnaire is voluntary, and responses will be collected anonymously to protect participants' privacy. Do you agree to participate in this survey?**

○ Agree

Personal and Demographic Information

2 **Sex**

○ Male ○ Female

3 **Age**

○ 18	○ 19	○ 20	○ 21	○ 22	○ 23
○ 24	○ 25	○ 26	○ 27	○ 28	○ 29
○ 30	○ 31	○ 32	○ 33	○ 34	○ 35

4 **Place of residence**

○ Urban area ○ Rural area

5 **Marital status**

○ Single ○ Married ○ Divorced/Widowed

6 **Income in Jordanian dinars**

○ 0-500 ○ 500-1,000 ○ 1,000-2,000 ○ More than 2,000

CONTINUED Personal and Demographic Information

7 University

- ○ Yarmouk University
- ○ Jordan University of Science and Technology
- ○ University of Jordan
- ○ Hashemite University
- ○ Al-Balqa Applied University
- ○ Mu'tah University
- ○ Al al-Bayt University
- ○ Al-Hussein Bin Talal University
- ○ Tafila Technical University
- ○ German Jordanian University
- ○ Al-Hussein Technical University
- ○ Private university

8 Field of study

- ○ Medical
- ○ Non-medical

9 Academic year

- ○ First year
- ○ Second year
- ○ Third year
- ○ Fourth year
- ○ Fifth year
- ○ Sixth year
- ○ Graduated

10 Academic performance

- ○ Excellent
- ○ Very good
- ○ Good
- ○ Acceptable/Pass

11 Do you have any chronic disease?

- ○ Yes
- ○ No

Attitudes and Choices in Managing Common Cold and Influenza

12 Which symptoms do you experience when you have a common cold and feel that you need treatment?
Select all that apply.

- ☐ Headache
- ☐ Fever
- ☐ Sore throat
- ☐ Muscle pain

13 If you prefer using treatment when you have a common cold or influenza, when do you start treatment after symptoms appear?

- ○ On the same day
- ○ After 1-2 days
- ○ After 3 days or more
- ○ I do not prefer using treatment

14 When you have a common cold or influenza, what is your first treatment choice?

- ○ Natural herbs (such as ginger, chamomile, thyme, or sage)
- ○ Medications (such as Panadol, decongestants, or antibiotics when needed)
- ○ A combination of both
- ○ I do not prefer any treatment and rely on rest

15 What is the main reason for choosing this type of treatment?
Select all that apply.

- ☐ Faster effectiveness
- ☐ Fewer side effects
- ☐ Lower cost
- ☐ Easy availability
- ☐ Successful personal experience

Use of Herbal Remedies

16 **Do you rely on herbal remedies when you are ill?**

- ○ Yes
- ○ No

17 **If you prefer using herbal remedies when you have a common cold, when do you start using them after symptoms appear?**

- ○ On the same day
- ○ After 1-2 days
- ○ After 3 days or more
- ○ I do not prefer using herbal remedies

18 **Which herbal remedies do you usually use when you have a common cold or influenza?**
Select all that apply.

- ☐ Ginger
- ☐ Chamomile
- ☐ Thyme
- ☐ Sage
- ☐ Anise
- ☐ Turmeric
- ☐ Mint
- ☐ I do not prefer using herbal remedies

19 **What motivates you to use herbal remedies for common cold symptoms?**
Select all that apply.

- ☐ Lack of time to visit a physician
- ☐ Long distance from home to clinics or hospitals
- ☐ Mild symptoms that are easy to treat
- ☐ High cost of visiting a physician
- ☐ Recommended by a pharmacist
- ☐ Recommended by friends or family
- ☐ Advertisements
- ☐ Previous experience
- ☐ I do not prefer using herbal remedies

20 **If natural herbs do not achieve the desired results, will you resort to treatment with medications?**

- ○ Yes, I will use medications
- ○ No, I will continue using herbs until I recover
- ○ I may combine herbs and medications
- ○ I do not prefer using herbal remedies

Use of Pharmaceutical Medications

21 **If you prefer using pharmaceutical treatments when you are ill, when do you start treatment after symptoms appear?**

- ○ On the same day
- ○ After 1-2 days
- ○ After 3 days or more
- ○ I do not prefer using treatments

CONTINUED Use of Pharmaceutical Medications

22
When you resort to medications, do you purchase them based on a prescription?

- ◯ Yes, I always consult a physician before purchasing any medication
- ◯ No, I purchase medications without a prescription based on my experience and knowledge
- ◯ I consult only a pharmacist
- ◯ I do not prefer using medications

23
Which medications do you usually use when you have a common cold or influenza?
Select all that apply.

- ☐ Analgesics (such as Panadol)
- ☐ Antibiotics (such as Amoclan)
- ☐ Decongestants (such as pseudoephedrine)
- ☐ Antihistamines
- ☐ I do not prefer using medications

24
Which factors influence your choice of pharmaceutical medications?
Select all that apply.

- ☐ Severity of symptoms
- ☐ Availability and ease of access
- ☐ Fewer side effects
- ☐ Physician recommendations
- ☐ I do not prefer using medications

25
What concerns you most about using medical medications?
Select all that apply.

- ☐ Possible side effects
- ☐ High cost
- ☐ Long-term dependency
- ☐ The body becoming resistant to medications
- ☐ I have no concerns about using them

QUESTION 26 Beliefs and Attitudes Toward Herbal Remedies and Medications

Please indicate how strongly you agree or disagree with each statement. Select one response in each row.

Statement	Strongly agree	Agree	Neutral	Disagree	Strongly disagree
1. I believe that my personal experience with using herbal remedies or medications is related to the experiences of people around me, including family, friends, and society.	○	○	○	○	○
2. The use of certain medicinal herbs as a key component in manufacturing some medications contributes to shaping society's attitudes toward modern medicine.	○	○	○	○	○
3. I stop using a particular medication for treating common cold symptoms when I hear about a medicinal herb that treats them.	○	○	○	○	○
4. If a physician recommends using herbal remedies, I would be more inclined to try them.	○	○	○	○	○
5. I believe that herbal remedies may be a complete alternative to medications.	○	○	○	○	○
6. I believe that medications are more effective than herbal treatments.	○	○	○	○	○
7. Herbal remedies have therapeutic effects equal to or greater than those of medications in cases of common cold.	○	○	○	○	○
8. I believe that medications are manufactured substances that weaken immunity compared with herbal treatments.	○	○	○	○	○
9. I believe that herbal remedies have no side effects compared with medications.	○	○	○	○	○
10. I believe that some herbal treatments may cause allergic reactions; therefore, they should be used cautiously and only after ensuring their suitability for the individual.	○	○	○	○	○

27 Where do you obtain information about the effectiveness of herbal remedies and medications in treating common cold and influenza?

Select all that apply.

- ☐ Academic sources / scientific books
- ☐ The internet and social media
- ☐ Physicians and pharmacists
- ☐ Personal experiences or recommendations from family and friends

Thank you for your participation. We appreciate your time and contribution to scientific research.
