# Supplementary material for: Herbal or conventional medicine? A cross-sectional study of Jordanian university students’ preferences for cold and flu treatment
Source: PLoS One. 2026 Sep 15;21(9):e0358179. doi: 10.1371/journal.pone.0358179 (PMC13577359; doi:10.1371/journal.pone.0358179)
Supplement: Supplementary file 4 [file pone.0358179.s004.pdf]

Supplementary File 4

Post-study Revised English Questionnaire

Herbal or Conventional Medicine? A Cross-Sectional Study of Jordanian University Students' Preferences for Cold and Flu Treatment

Important transparency note: This revised English questionnaire was prepared after completion of data collection in response to reviewer feedback and for potential future use. It was not administered to participants in the present study. The study data were collected using the original Arabic questionnaire; the original English translation is provided separately.

This questionnaire examines university students' preferences and self-reported practices regarding herbal remedies and pharmaceutical treatments for common cold and influenza symptoms. Completion takes approximately five minutes. Participation is voluntary, responses are anonymous, and all information is used solely for research purposes.

Consent

1. Filling out this questionnaire is voluntary, and responses will be collected anonymously to protect participants' privacy. Do you agree to participate in this survey?

Select one response.

- ○ Agree

Personal and Demographic Information

2. Sex

Select one response.

- ○ Male
- ○ Female

3. Age (years)

Select one response.

- ○ 18
- ○ 19
- ○ 20
- ○ 21
- ○ 22
- ○ 23
- ○ 24
- ○ 25
- ○ 26
- ○ 27
- ○ 28
- ○ 29
- ○ 30
- ○ 31
- ○ 32
- ○ 33
- ○ 34
- ○ 35

4. Place of residence

Select one response.

- ○ Urban area
- ○ Rural area

5. Marital status

Select one response.

- ○ Single
- ○ Married
- ○ Divorced/Widowed

6. What is your average monthly personal income or financial support available to you, including family support and income from part-time employment (in Jordanian dinars)?

Select one response.

- ○ 0-500
- ○ 501-1,000
- ○ 1,001-2,000
- ○ More than 2,000

7. University

Select one response.

- ○ Yarmouk University
- ○ Jordan University of Science and Technology
- ○ University of Jordan
- ○ Hashemite University
- ○ Al-Balqa Applied University
- ○ Mu'tah University
- ○ Al al-Bayt University
- ○ Al-Hussein Bin Talal University
- ○ Tafila Technical University
- ○ German Jordanian University
- ○ Al-Hussein Technical University
- ○ Private university

8. Field of study

Select one response.

- ○ Medical
- ○ Non-medical

9. Academic year

Select one response.

- ○ First year
- ○ Second year
- ○ Third year
- ○ Fourth year
- ○ Fifth year
- ○ Sixth year
- ○ Expected to graduate (currently enrolled with six or fewer credit hours remaining)

10. Academic performance

Select one response.

- ○ Excellent
- ○ Very good
- ○ Good
- ○ Acceptable/Pass

11. Do you have any chronic disease?

Select one response.

- ○ Yes
- ○ No

Previous Experience and Treatment Choices for Common Cold and Influenza

S1. Have you ever experienced symptoms that you considered to be a common cold or influenza?

Select one response. If No, proceed to Question 26.

- ○ Yes
- ○ No

12. Based on your previous experiences with common cold or influenza, which symptoms usually made you feel the need for treatment?

Select all that apply.

- □ Headache
- □ Fever
- □ Sore throat
- □ Muscle pain

13. Based on your usual practice, when did you generally begin treatment after symptoms appeared?

Select one response.

- ○ On the same day
- ○ After 1-2 days
- ○ After 3 days or more
- ○ I did not usually use any treatment

14. Based on your usual practice, what was your typical first treatment choice when experiencing common cold or influenza symptoms?

Select one response.

- ○ Herbal remedies (such as ginger, chamomile, thyme, or sage)
- ○ Pharmaceutical treatments (such as paracetamol, decongestants, or antibiotics when clinically indicated)
- ○ A combination of herbal remedies and pharmaceutical treatments
- ○ No treatment; I relied on rest

15. What were the main reasons for choosing that treatment approach?

Select all that apply.

- □ Faster effectiveness
- □ Fewer side effects
- □ Lower cost
- □ Easy availability
- □ Positive personal experience

Use of Herbal Remedies

16. Based on your previous experiences, did you use herbal remedies when you were ill?

Select one response.

- ○ Yes
- ○ No

17. If you used herbal remedies, when did you usually begin using them after symptoms appeared?

Select one response.

- ○ On the same day
- ○ After 1-2 days
- ○ After 3 days or more
- ○ I did not use herbal remedies

18. Which herbal remedies did you typically use for common cold or influenza symptoms?

Select all that apply.

- □ Ginger
- □ Chamomile
- □ Thyme
- □ Sage
- □ Anise
- □ Turmeric
- □ Mint
- □ I did not use herbal remedies
- □ Other (please specify): ________________________________

19. What motivated you to use herbal remedies for common cold or influenza symptoms?

Select all that apply.

- □ Lack of time to visit a doctor
- □ Distance to clinics or hospitals
- □ Mild symptoms that were easy to treat
- □ High cost of doctor visits
- □ Recommendation from a pharmacist
- □ Recommendation from family or friends
- □ Advertisements
- □ Previous experience
- □ I did not use herbal remedies
- □ Other (please specify): ________________________________

20. If herbal remedies did not achieve the desired symptom relief, what did you usually do?

Select one response.

- ○ Switched to pharmaceutical treatments
- ○ Continued using herbal remedies until recovery
- ○ Combined herbal remedies and pharmaceutical treatments
- ○ I did not use herbal remedies

Use of Pharmaceutical Treatments

21. If you used pharmaceutical treatments, when did you usually begin using them after symptoms appeared?

Select one response.

- ○ On the same day
- ○ After 1-2 days
- ○ After 3 days or more
- ○ I did not use pharmaceutical treatments

22. When obtaining pharmaceutical treatments, which option best described your usual practice?

Select one response.

- ○ I consulted a doctor and used treatment based on a prescription
- ○ I purchased pharmaceutical treatments without a prescription based on my own experience and knowledge
- ○ I consulted only a pharmacist
- ○ I did not use pharmaceutical treatments

23. Which pharmaceutical treatments did you typically use for common cold or influenza symptoms?

Select all that apply.

- □ Analgesics/antipyretics (such as paracetamol)
- □ Antibiotics
- □ Decongestants (such as pseudoephedrine)
- □ Antihistamines
- □ I did not use pharmaceutical treatments
- □ Other (please specify): ______________________________

24. Which factors influenced your choice of pharmaceutical treatments?

Select all that apply.

- □ Severity of symptoms
- □ Availability and ease of access
- □ Fewer side effects
- □ Doctor recommendations
- □ I did not use pharmaceutical treatments
- □ Other (please specify): ______________________________

25. What concerned you most about using pharmaceutical treatments?

Select all that apply.

- □ Possible side effects
- □ High cost
- □ Long-term dependency
- □ Reduced effectiveness due to repeated use
- □ I had no concerns about using them
- □ Other (please specify): ______________________________

Beliefs and Attitudes Toward Herbal Remedies and Pharmaceutical Treatments

26. Please indicate how strongly you agree or disagree with each statement.

Statement	Strongly agree	Agree	Neutral	Disagree	Strongly disagree
1. My personal experience with herbal remedies or pharmaceutical treatments is influenced by the experiences of people around me, including family, friends, and the wider community.	○	○	○	○	○
2. The use of medicinal herbs as ingredients in some pharmaceutical products contributes to shaping community attitudes toward modern medicine.	○	○	○	○	○
3. I stop using a pharmaceutical treatment for common cold symptoms when I hear about a herbal remedy that may treat those symptoms.	○	○	○	○	○
4. If a physician recommends using herbal remedies, I am more willing to try them.	○	○	○	○	○
5. Herbal remedies can be a complete alternative to pharmaceutical treatments.	○	○	○	○	○

Question 26 (continued)

Statement	Strongly agree	Agree	Neutral	Disagree	Strongly disagree
6. Pharmaceutical treatments are more effective than herbal remedies.	○	○	○	○	○
7. Herbal remedies may have a healing ability equal to or greater than that of pharmaceutical treatments for common cold symptoms.	○	○	○	○	○
8. Pharmaceutical treatments are manufactured substances that weaken the immune system compared with herbal remedies.	○	○	○	○	○
9. Herbal remedies have no side effects compared with pharmaceutical treatments.	○	○	○	○	○
10. Some herbal remedies may cause allergic reactions; therefore, they should be used cautiously after ensuring their suitability for the individual.	○	○	○	○	○

27. Where did you obtain information about the effectiveness of herbal remedies and pharmaceutical treatments for common cold or influenza symptoms?

Select all that apply.

- ◻ Academic sources or scientific books
- ◻ Doctors and pharmacists
- ◻ The internet and social media
- ◻ Personal experience or recommendations from family and friends
- ◻ Other (please specify): ______________________________

Thank you for your participation.
