# Supplementary material for: Herbal or conventional medicine? A cross-sectional study of Jordanian university students’ preferences for cold and flu treatment
Source: PLoS One. 2026 Sep 15;21(9):e0358179. doi: 10.1371/journal.pone.0358179 (PMC13577359; doi:10.1371/journal.pone.0358179)
Supplement: Supplementary file 5 [file pone.0358179.s005.pdf]

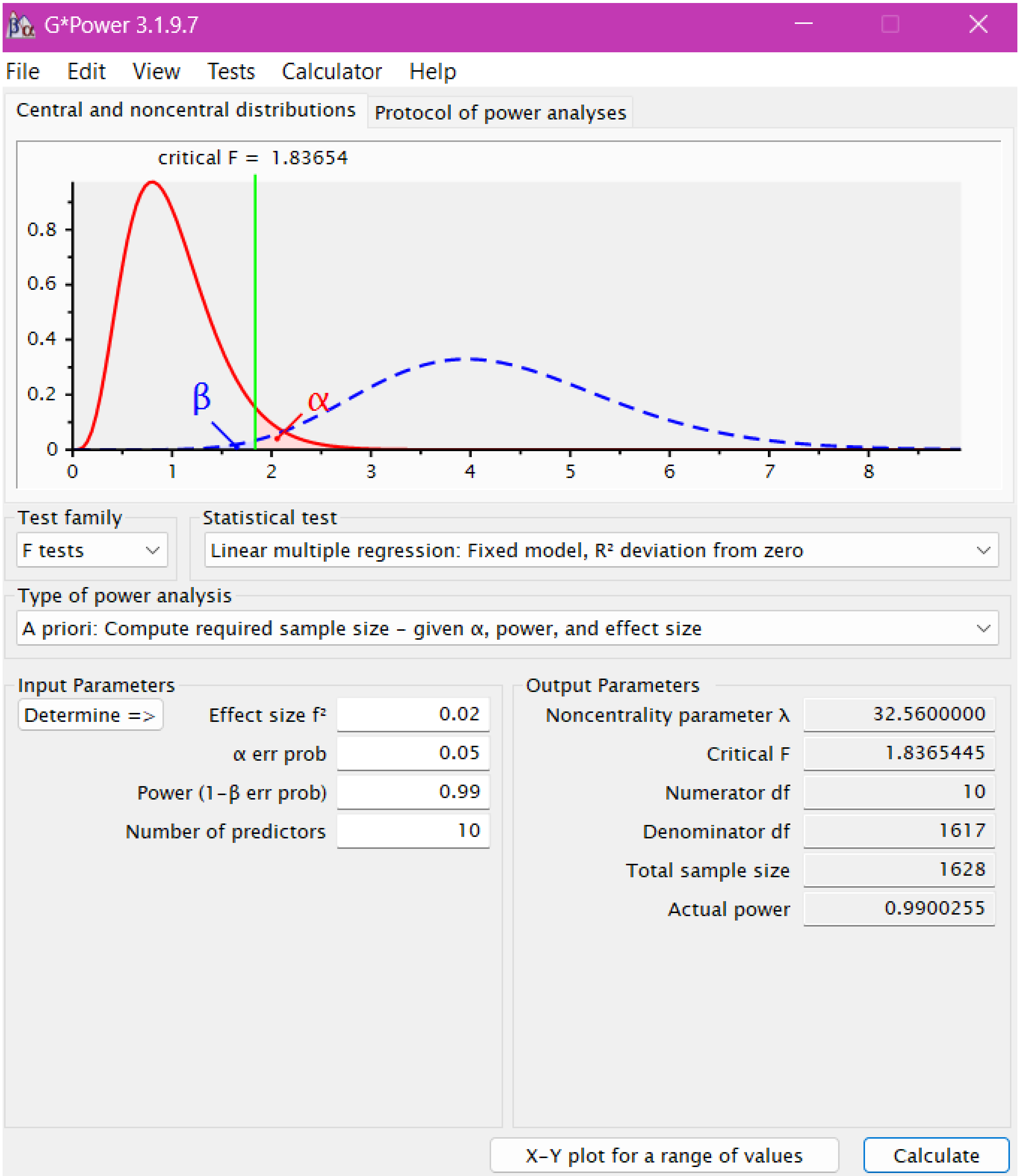
G*Power 3.1.9.7
File Edit View Tests Calculator Help
Central and noncentral distributions
Protocol of power analyses
critical F = 1.83654
β
α
Test family
F tests
Statistical test
Linear multiple regression: Fixed model, R² deviation from zero
Type of power analysis
A priori: Compute required sample size – given α, power, and effect size
Input Parameters
Determine =>
Effect size f² 0.02
α err prob 0.05
Power (1-β err prob) 0.99
Number of predictors 10
Output Parameters
Noncentrality parameter λ 32.5600000
Critical F 1.8365445
Numerator df 10
Denominator df 1617
Total sample size 1628
Actual power 0.9900255
X-Y plot for a range of values
Calculate

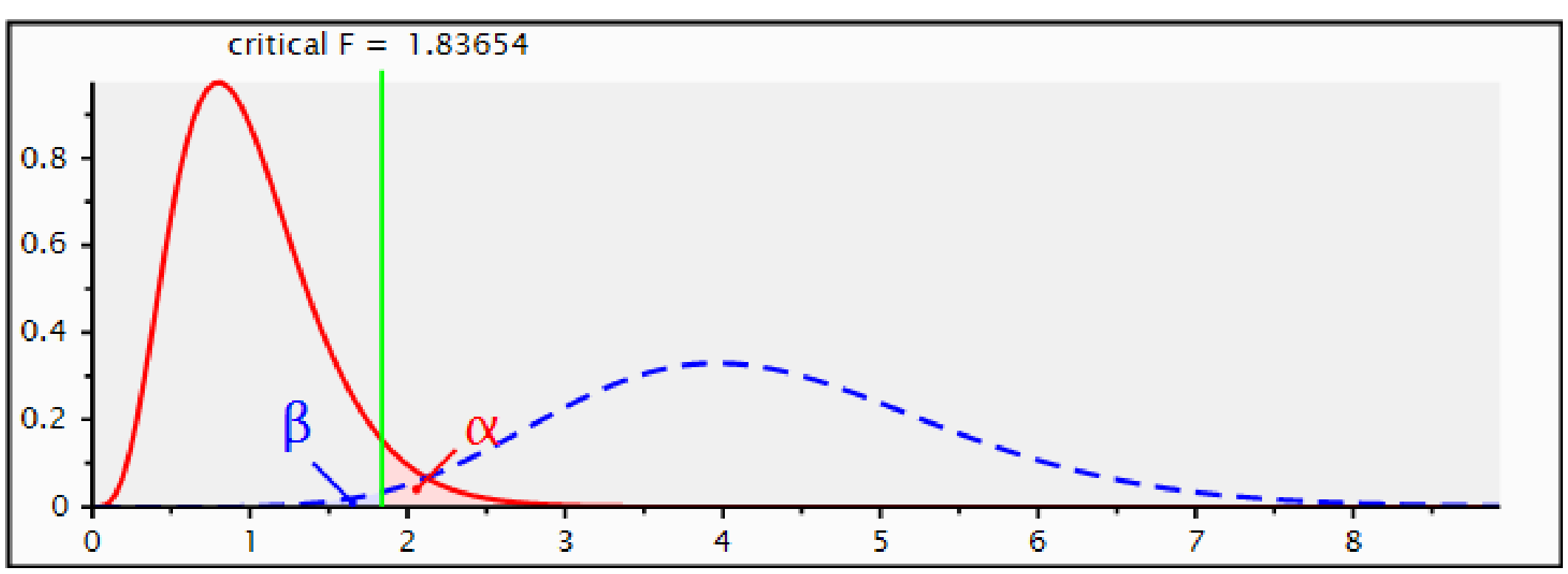
critical F = 1.83654
β
α

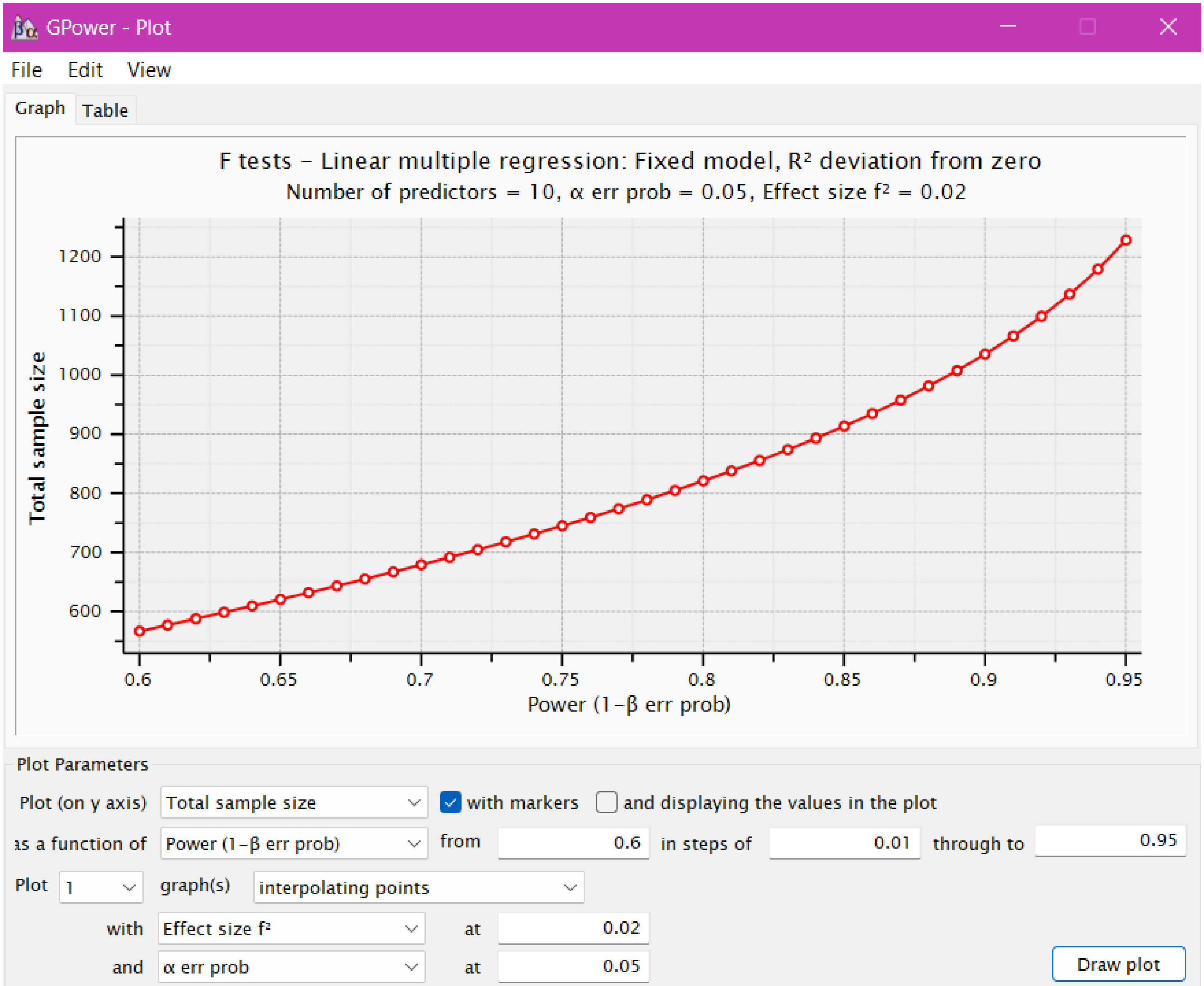
GPower - Plot
File
Edit
View
Graph
Table
F tests – Linear multiple regression: Fixed model, R² deviation from zero
Number of predictors = 10, α err prob = 0.05, Effect size f² = 0.02
Total sample size
1200
1100
1000
900
800
700
600
0.6
0.65
0.7
0.75
0.8
0.85
0.9
0.95
Power (1–β err prob)
Plot Parameters
Plot (on y axis)
Total sample size
with markers
and displaying the values in the plot
as a function of
Power (1–β err prob)
from
0.6
in steps of
0.01
through to
0.95
Plot
1
graph(s)
interpolating points
with
Effect size f²
at
0.02
and
α err prob
at
0.05
Draw plot

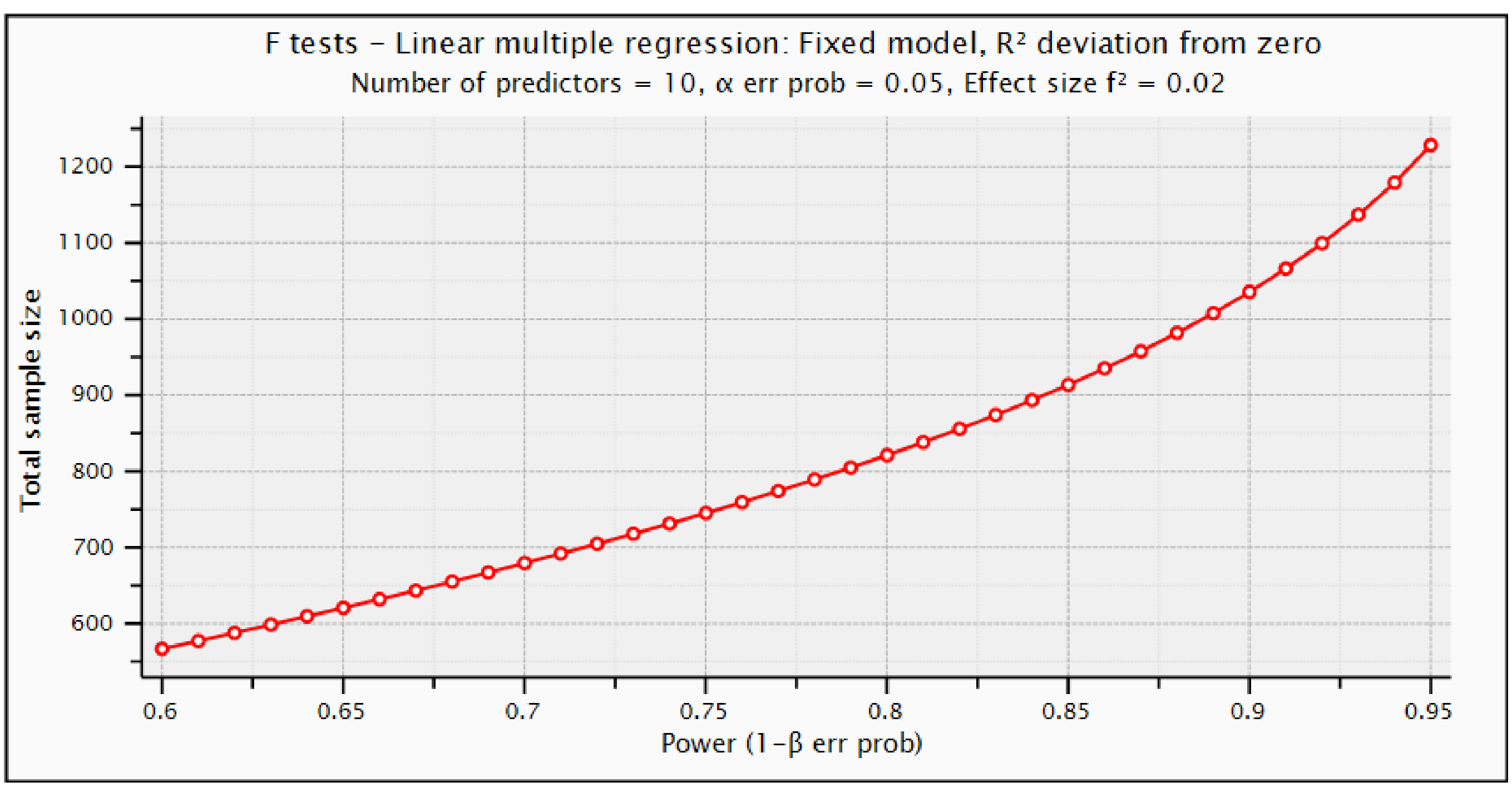
F tests – Linear multiple regression: Fixed model, R² deviation from zero
Number of predictors = 10, α err prob = 0.05, Effect size f² = 0.02
Total sample size
1200
1100
1000
900
800
700
600
0.6
0.65
0.7
0.75
0.8
0.85
0.9
0.95
Power (1–β err prob)
