# Supplementary material for: Herbal or conventional medicine? A cross-sectional study of Jordanian university students’ preferences for cold and flu treatment
Source: PLoS One. 2026 Sep 15;21(9):e0358179. doi: 10.1371/journal.pone.0358179 (PMC13577359; doi:10.1371/journal.pone.0358179)
Supplement: Supplementary file 6 [file pone.0358179.s006.pdf]

Supplementary File 6. Supplementary Tables

Supplementary Table S1. Multinomial logistic regression for first-line treatment choice for common cold and influenza after excluding participants from Yarmouk University.

Reference category: Do not use any treatment.

No.	Variable	Category / comparison	Herbal remedies		Pharmaceutical treatments		Combined therapy	
			aOR (95% CI)	p-value	aOR (95% CI)	p-value	aOR (95% CI)	p-value
1	Age	Continuous (per year)	1.05 (0.96–1.15)	0.268	0.95 (0.86–1.05)	0.285	1.02 (0.94–1.12)	0.620
2	Sex	Female (Baseline: Male)	1.82 (0.93–3.56)	0.080	1.40 (0.70–2.78)	0.339	**2.49 (1.30–4.77)**	**0.006**
3	Residence	Rural (Baseline: Urban)	0.60 (0.31–1.14)	0.118	**0.40 (0.20–0.80)**	**0.009**	**0.43 (0.23–0.80)**	**0.008**
4	Field of study	Medical (Baseline: non-medical)	1.24 (0.74–1.41)	0.296	0.98 (0.50–1.92)	0.953	1.47 (0.78–2.77)	0.230
5	Presence of chronic diseases	Suffering (Baseline: no chronic disease)	1.00 (0.31–2.18)	0.993	1.29 (0.59–2.85)	0.526	1.68 (0.80–3.51)	0.169
6	Symptoms type	Headache	1.73 (0.89–3.35)	0.107	1.66 (0.84–3.31)	0.146	1.88 (0.99–3.56)	0.054
		Fever	0.97 (0.50–1.88)	0.924	1.37 (0.69–2.72)	0.367	1.19 (0.63–2.26)	0.591
		Sore throat	1.74 (0.87–3.40)	0.102	**2.50 (1.24–5.08)**	**0.011**	**2.67 (1.39–5.12)**	**0.003**
		Muscle pain	0.56 (0.29–1.08)	0.083	0.63 (0.32–1.25)	0.186	0.87 (0.46–1.66)	0.681

Note. aOR, adjusted odds ratio; CI, confidence interval. Bold values indicate statistically significant associations at $p < 0.05$.

Supplementary Table S1 (continued).

No.	Variable	Category / comparison	Herbal remedies		Pharmaceutical treatments		Combined therapy	
			aOR (95% CI)	p-value	aOR (95% CI)	p-value	aOR (95% CI)	p-value
7	Concern about medication side effects	Possible side effects	1.32 (0.66–2.62)	0.436	0.83 (0.41–1.69)	0.604	0.97 (0.50–1.89)	0.925
		Long-term dependency	**0.29 (0.13–0.63)**	**0.002**	**0.32 (0.14–0.72)**	**0.006**	**0.32 (0.15–0.68)**	**0.003**
		No concerns	**0.21 (0.08–0.57)**	**0.002**	**0.28 (0.10–0.78)**	**0.015**	**0.29 (0.11–0.74)**	**0.010**

Note. aOR, adjusted odds ratio; CI, confidence interval. Bold values indicate statistically significant associations at $p < 0.05$.

Supplementary Table S2. Pattern matrix for the refined 8-item belief scale.

No.	Item	Factor 1	Factor 2
1	I believe herbal remedies can be a complete alternative to medications	0.84	—
2	Herbal remedies may have therapeutic effects equal to or greater than medications in some common cold/influenza situations	0.79	—
3	I stop using a pharmaceutical treatment for common cold symptoms when I hear about a herbal remedy that may treat those symptoms.	0.58	—
4	I believe that pharmaceutical treatments are manufactured substances that weaken the immune system compared with herbal remedies.	0.66	—
5	I believe herbal remedies have no side effects compared with medications	0.49	—
6	My personal experience with herbal remedies or pharmaceutical treatments is influenced by the experiences of people around me, including family, friends, and the wider community.	—	0.69
7	The use of some herbal remedies may shape community attitudes toward modern medicine	—	0.64
8	If a physician recommended herbal remedies, I would be more willing to try them	—	0.48

Note. Loadings <0.30 are suppressed. Extraction method: principal axis factoring; rotation method: direct oblimin with Kaiser normalization.

Supplementary Table S3. Descriptive statistics for all independent variables included in the multinomial logistic regression models.

Variable	Category or summary	n / Summary	%
**Demographic and clinical variables**			
Age (years)	Mean ± SD	20.87 ± 3.02	—
Sex	Male	540	30.1
	Female	1,257	69.9
Residence	Urban	1,127	62.7
	Rural	670	37.3
Field of study	Non-medical	910	50.6
	Medical	887	49.4
Presence of chronic disease	No	1,341	74.6
	Yes	456	25.4
University group	Yarmouk University	1,138	63.3
	Other universities	659	36.7
**Symptoms prompting treatment**			
Headache	Yes	1,148	63.9
	No	649	36.1
Fever	Yes	971	54.0
	No	826	46.0
Sore throat	Yes	1,271	70.7
	No	526	29.3
Muscle pain	Yes	901	50.1
	No	896	49.9

Supplementary Table S3 (continued).

Variable	Category or summary	n / Summary	%
**Concerns about pharmaceutical treatments**			
Possible side effects	Yes	978	54.4
	No	819	45.6
Long-term dependency	Yes	861	47.9
	No	936	52.1
No concerns	Yes	270	15.0
	No	1,527	85.0
**Medication-obtaining predictor**			
Use of pharmaceutical treatments based on a doctor's prescription	No	1,060	59.0
	Yes	737	41.0
**Belief-related predictors**			
I believe that herbal remedies can be a complete alternative to pharmaceutical treatments	Strongly agree	328	18.3
	Agree	401	22.3
	Neutral	498	27.7
	Disagree	422	23.5
	Strongly disagree	148	8.2
Herbal remedies have a healing ability that equals or exceeds that of pharmaceutical treatments in cases of colds	Strongly agree	308	17.1
	Agree	508	28.3
	Neutral	623	34.7
	Disagree	295	16.4
	Strongly disagree	63	3.5

Supplementary Table S3 (continued).

Variable	Category or summary	n / Summary	%
**Belief-related predictors (continued)**			
I believe that pharmaceutical treatments are manufactured substances that weaken the immune system compared with herbal remedies	Strongly agree	385	21.4
	Agree	543	30.2
	Neutral	478	26.6
	Disagree	303	16.9
	Strongly disagree	88	4.9
I believe that herbal remedies have no side effects compared with pharmaceutical treatments	Strongly agree	473	26.3
	Agree	613	34.1
	Neutral	413	23.0
	Disagree	236	13.1
	Strongly disagree	62	3.5

Note. Percentages are based on N = 1,797 and are presented to one decimal place. Age is presented as mean ± standard deviation. Symptoms and medication-related concerns originated from multiple-response items and were coded as separate binary predictors. The doctor-prescription variable was coded as Yes only for participants who reported always consulting a doctor before purchasing medications; all other medication-obtaining responses were coded as No.

Supplementary Table S4. Association between sex and first-line treatment choice for common cold and influenza.

No.	Variable	Category	First-line treatment choice for common cold and influenza				Total n (%)	p-value
			Herbal remedies n (%)	Pharmaceutical treatments n (%)	Combined therapy n (%)	Do not use any treatment n (%)		
**1**	**Sex**	Male	148 (27.4)	131 (24.3)	195 (36.1)	66 (12.2)	540 (100.0)	**<0.001**
		Female	328 (26.1)	244 (19.4)	605 (48.1)	80 (6.4)	1,257 (100.0)	

Note. Values are presented as n (row %). Percentages are shown to one decimal place. The p-value was obtained using the chi-square test.

Supplementary Table S5. Exploratory factor analysis and internal consistency of the refined 8-item belief scale.

No.	Parameter	Result
**1**	Initial number of belief items	10
**2**	Final number of retained items	8
**3**	Extraction method	Principal axis factoring
**4**	Rotation method	Direct oblimin with Kaiser normalization
**5**	Kaiser-Meyer-Olkin measure	0.87
**6**	Bartlett's test of sphericity, χ^2(df)	4,609.92 (28)
**7**	Bartlett's test p-value	$p < 0.001$
**8**	Number of retained factors	2
**9**	Total variance explained	59.9%
**10**	Correlation between factors	0.64
**11**	Cronbach's alpha, Factor 1 (5 items)	0.83
**12**	Cronbach's alpha, Factor 2 (3 items)	0.66

Note. Two items (items 6 and 10 of Question 26) from the initial 10-item belief domain were excluded because of weaker psychometric performance during scale refinement. The final 8-item solution demonstrated a clearer two-factor structure, representing (1) pro-herbal efficacy and safety beliefs and (2) experience- and social influence-related beliefs.
